# Comparative genome analysis of entomopathogenic fungi reveals a complex set of secreted proteins

**DOI:** 10.1186/1471-2164-15-822

**Published:** 2014-09-29

**Authors:** Charley Christian Staats, Ângela Junges, Rafael Lucas Muniz Guedes, Claudia Elizabeth Thompson, Guilherme Loss de Morais, Juliano Tomazzoni Boldo, Luiz Gonzaga Paula de Almeida, Fábio Carrer Andreis, Alexandra Lehmkuhl Gerber, Nicolau Sbaraini, Rana Louise de Andrade da Paixão, Leonardo Broetto, Melissa Landell, Lucélia Santi, Walter Orlando Beys-da-Silva, Carolina Pereira Silveira, Thaiane Rispoli Serrano, Eder Silva de Oliveira, Lívia Kmetzsch, Marilene Henning Vainstein, Ana Tereza Ribeiro de Vasconcelos, Augusto Schrank

**Affiliations:** Centro de Biotecnologia, Universidade Federal do Rio Grande do Sul (UFRGS), P. O. Box 15005, Porto Alegre, RS CEP 91501-970 Brazil; Laboratório Nacional de Computação Científica (LNCC), Av. Getúlio Vargas, 333, Petrópolis, RJ Brazil

**Keywords:** Genome sequence, Entomopathogenic fungi, Secretome, Phylogenomics

## Abstract

**Background:**

*Metarhizium anisopliae* is an entomopathogenic fungus used in the biological control of some agricultural insect pests, and efforts are underway to use this fungus in the control of insect-borne human diseases. A large repertoire of proteins must be secreted by *M. anisopliae* to cope with the various available nutrients as this fungus switches through different lifestyles, i.e., from a saprophytic, to an infectious, to a plant endophytic stage. To further evaluate the predicted secretome of *M. anisopliae*, we employed genomic and transcriptomic analyses, coupled with phylogenomic analysis, focusing on the identification and characterization of secreted proteins.

**Results:**

We determined the *M. anisopliae* E6 genome sequence and compared this sequence to other entomopathogenic fungi genomes. A robust pipeline was generated to evaluate the predicted secretomes of *M. anisopliae* and 15 other filamentous fungi, leading to the identification of a core of secreted proteins. Transcriptomic analysis using the tick *Rhipicephalus microplus* cuticle as an infection model during two periods of infection (48 and 144 h) allowed the identification of several differentially expressed genes. This analysis concluded that a large proportion of the predicted secretome coding genes contained altered transcript levels in the conditions analyzed in this study. In addition, some specific secreted proteins from *Metarhizium* have an evolutionary history similar to orthologs found in *Beauveria*/*Cordyceps.* This similarity suggests that a set of secreted proteins has evolved to participate in entomopathogenicity.

**Conclusions:**

The data presented represents an important step to the characterization of the role of secreted proteins in the virulence and pathogenicity of *M. anisopliae.*

**Electronic supplementary material:**

The online version of this article (doi:10.1186/1471-2164-15-822) contains supplementary material, which is available to authorized users.

## Background

It is estimated that over 600,000 species of fungi exist, and it is assumed that these species can be found in almost all habitats on Earth. However, only a few of these species have been described [1]. Most fungal species have developed saprophytic interactions in soil and in water or in association with mycorrhizal plants, as either arbuscular mycorrhizae or ectomycorrhizae. Moreover, fungal species are known to cause disease in several hosts, including mammals, arthropods, and plants [2]. To adapt to such a large variety of habitats, fungi have developed a prolific capability to export proteins to the extracellular space as an important mechanism to acquire nutrients [3]. Therefore, secretomes, which are defined as the global set of proteins produced by a cell and exported to the extracellular space in a determined time and condition, represent an important target for understanding the mechanisms of fungal adaptation. For instance, both saprophytic and pathogenic fungi must quickly adapt to variations in carbon and nitrogen availability. Because fungi generally obtain nutrients from the digestion of extracellular polymers, such as cellulose and chitin, fungi must produce copious amounts of extracellular enzymes to allow for the efficient hydrolysis of biopolymers during the infection process or from their natural environment [3].

A diverse group of fungi is associated with arthropods, the largest class of eukaryotic species on Earth, and plays a role in controlling their populations, in particular of insects [4]. The most well known insect-associated fungi are entomopathogens, which are necrotrophic fungi that actively penetrate the host exoskeleton and proliferate in the hemocoel until all internal tissues have been degraded. The infection process of entomopathogenic fungi depends on the secretion of a plethora of enzymes and toxins, which serve to penetrate and kill the host, as well as to provide nutrients through the action of biopolymer-degrading enzymes [5–10]. The best-characterized example of a relation between an entomopathogenic fungus and its hosts is the genus *Metarhizium*. Several lines of evidence suggest that the infection cycle of *Metarhizium* can be schematically divided into the following steps: (i) conidia adherence to the host cuticle through hydrophobic interactions and thin mucilaginous material; (ii) conidia germination and development; (iii) germ-tube differentiation into appressoria; (iv) cuticle penetration; (v) hyphae differentiation into blastospores/hyphal bodies in the hemolymph; (vi) host colonization; (vii) extrusion to the host cadaver surface; and (viii) conidiophore formation and conidia production [11]. The participation of many proteins, including secreted proteins, has been described for the infection process (reviewed in [12]). More recently, the existence of alternative mechanisms has been suggested during the control of *Aedes aegypti*, the mosquito vector of dengue and yellow fever [13]. Because different *Metarhizium* species can infect and kill more than 200 species from 50 insect and arthropod families [14], some isolates have been widely used as bioagents to control a wide variety of pests [15]. Indeed, almost 50 different formulations employing *Metarhizium* are commercially available [16].

In fact, the *Metarhizium* species generally regarded as *M. anisopliae* is composed of nine different species, which can be most frequently isolated from either soil or insects [17]. The genomes of the *M. anisopliae* ARSEF 23, which are currently classified as *M. robertsii*[17], a broad-spectrum insect pathogen, and of the acridid-specific *M. acridum* CQMa 102, were characterized [18]. The sequence analysis of these genomes revealed that they are highly syntenic and possess many genes that allow for the different lifestyles of *Metarhizium* spp*.* In addition, a phylogenomic analysis showed that *M. robertsii* and *M. acridum* are more related to plant endophytes and pathogens than to animal pathogens. Moreover, this analysis showed that the sequenced genome was from *M. robertsii*, which had been misclassified as *M. anisopliae*[18]. Further information concerning the evolution of entomopathogenic fungi originates from the characterization of the entomopathogen *Beauveria bassiana* genome, which contributed to the identification of a common set of gene families potentially associated with fungal entomopathogenicity [19].

The large collection of fungal genomes sequenced, including entomopathogens, plant pathogens, mycopathogens, and mammal pathogens, allows the shared and exclusive genes present in the predicted secretomes of fungal species to be identified. To analyze the importance of secreted proteins in the virulence of fungal pathogens, we sequenced the genome of *M. anisopliae* strain E6 and performed a comparative study of this genome, emphasizing the predicted secretome among distinct fungal species.

## Results and discussion

### General features and comparative analyses of the *M. anisopliae*E6 genome

The genome sequence of *Metarhizium anisopliae* E6 was obtained using a 454-based pyrosequencing approach, with 19-fold genome coverage. The assembly performed using Newbler 2.8 and WGS-CA 7.0 software resulted in 191 and 516 scaffolds with 38,326,054 and 38,454,426 bp, respectively (Table 1). The longest scaffold identified using Newbler software was 1,756,362 bp, whereas the longest scaffold identified using WGS-CA software was 638,367 bp. Using Newbler and WGS-CA software, the N50 scaffold size was calculated as 622.80 kb and as 167.52 kb and the N50 contig size was measured as 157.96 kb and as 125.69 kb, respectively. Both assemblies were combined using the assembly tool Minimus2, and the total *M. anisopliae* genome size was 38.5 Mb (Table 1). In comparison to other entomopathogenic fungal genomes, *M. anisopliae* E6 has a genome size similar to *M. robertsii* ARSEF 23 and to *M. acridum* CQMa 102 and is larger than the *Cordyceps militaris* CM01 and *B. bassiana* ARSEF 2860 genomes (Table 2). The GC content of *M. anisopliae* E6 is 51%, which is similar to the values reported for *M. robertsii*, *M. acridum*, *C. militaris* and *B. bassiana*, which are fungi traditionally compared with *M. anisopliae*. The *M. anisopliae* E6 gene density is 280 genes per Mbp, which is extremely similar to the values found for the fungi previously cited, with an average of 2.7 exons per gene. The number of transfer RNAs (tRNAs) identified in *M. anisopliae* E6 was 181, which is higher than those tRNAs found in other fungi (Table 2). This finding may reflect the use of different methodologies for analyzing other fungal genomes [18, 19], which were assessed using a combination of different predictors, including tRNAScan-SE [20] and Infernal [21].

**Table 1:** **General information concerning the** ***M. anisopliae*** **E6 genome assembly**

Software	Newbler 2.8	WGS-CA 7.0	Minimus2/Consed
Scaffolds	191	516	-
Total scaffold size (bp)	38,326,054	38,454,426	-
Contigs	677	688	376^*^
Total contig size (bp)	38,369,953	38,434,596	38,478,534
Scaffold N50	622,803	167,523	-
Contig N50	157,963	125,686	319,537
Longest scaffold	1,756,362	638,367	-
Longest contig	735,175	638,367	1,044,648
Coverage	19 X	19.6 X	-
Singlets	3,552	39,616	-

^*^A total of 366 contigs larger than 200 base pairs were deposited in NCBI with the accession number JNNZ00000000.

**Table 2:** **Comparison of the primary genome features between** ***M. anisopliae*** **and other entomopathogenic fungi**

Feature	***M. anisopliae***	***M. robertsii***	***M. acridum***	***C. militaris***	***B. bassiana***
Strain	E6	ARSEF23	CQMa102	CM01	ARSEF2860
Host-range	Broad	Broad	Locust	Broad	Broad
Sequencing plataform	454	Solexa	Solexa	454/Illumina	454/Illumina
Size (Mbp)	38.5	39.0	38.1	32.2	33.7
Coverage	19 X	100 X	107 X	147 X	76.7 X
GC content (%)	51.0	51.5	50.0	51.4	51.5
Protein-coding genes	10,817	10,582	9,849	9,684	10,366
Gene density*	280	271	259	301	308
Exons/gene	2.6	2.8	2.7	3.0	2.7
tRNA	181	141	122	136	113

*M. robertsii* and *M. acridum* data [18], as well from *B. bassiana*[19] and *C. militaris*[22] were collected from the respectives references.

*Genes per Mbp.

**Figure 1:**
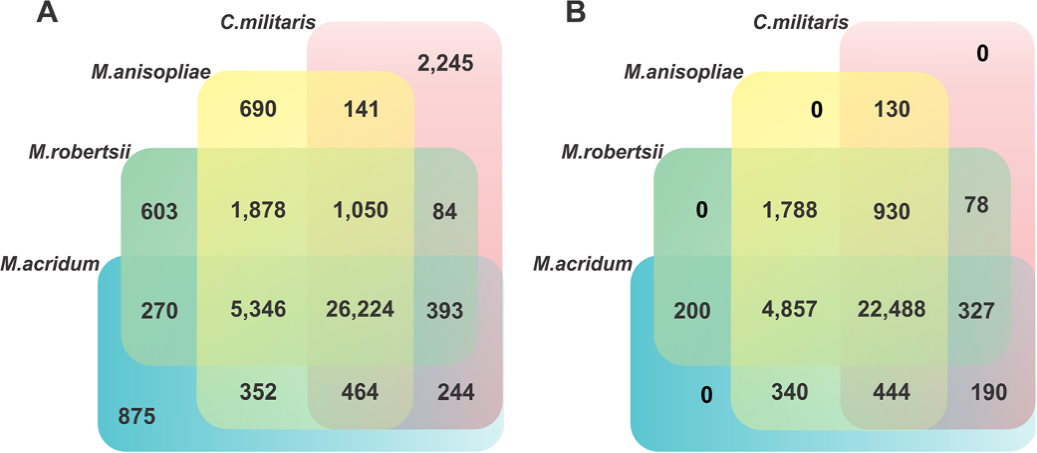
**Comparative genomics analyses of entomopathogenic fungi.** Venn diagram comparing the complete proteomes **(A)** or the ortholog sequences alone **(B)**. *M. anisopliae* (*M. anisopliae* E6), *M. robertsii* (*M. anisopliae* ARSEF 23), *M. acridum* (*M. acridum* CQMa 102) and *C. militaris* (*C. militaris* CM01) were used for comparisons. The diagram in B was constructed using best bidirectional BLAST hits, >60% positive amino acid alignments, >60% subject coverage and a cut-off e-value of <1e-05, without paralogs.

The genome was predicted to have 10,817 protein-coding genes, a value slightly higher than those values found for *M. robertsii* (10,582), *M. acridum* (9,849) and *C. militaris* (9,684) (Table 2). There are 3,820 hypothetical proteins in *M. anisopliae* E6, which account for 35.3% of its genome. Based on comparisons with previously cited fungi, *M. anisopliae* E6 has 690 (6.4%) exclusive protein coding genes (Figure 1A). Both host-generalist fungi *M. anisopliae* E6 and *M. robertsii* show a similar number of exclusive (species-specific) genes. However, the specialist fungus *M. acridum* has a significantly higher number of exclusive genes (875), and *C. militaris*, the teleomorphic species, has the highest number (2,245) of exclusive genes (Figure 1A) among these entomopathogens. Considering the four fungi, 62.64% (26,224/41,859) of all proteins are shared. As expected, *M. anisopliae* shares more proteins exclusively with *M. robertsii* (1,878) than with *M. acridum* (352) or with *C. militaris* (141). By excluding all paralogous genes present in each fungal genome, we could analyze the distribution of orthologous genes among the four fungi that presented 1:1 ortholog genes, representing 86% (22,488/26,224) of the shared genes (Figure 1B). BLAST and OrthoMCL analyses were conducted to evaluate the presence of ortholog sequences in *Metarhizium* spp. and showed that 94.2% of the *M. anisopliae* E6 coding sequences display matches in *M. robertsii* and 90.8% display matches in *M. acridum.* A broader comparison with the sequences of 16 additional fungal genomes revealed that 592 sequences are unique to *M. anisopliae* E6. Thus, increasing the number of fungal genomes from 4 to 16 from the different lifestyles does not significantly diminish the number of exclusive proteins, which are largely classified as hypothetical (97.5%), suggesting that these proteins may develop specific roles in *M. anisopliae* that differ from counterparts *M. acridum* and *M. robertsii.*

### Comparative analysis of genes involved in pathogen-host interactions

To evaluate the presence of genes known and suggested to be involved in pathogenic and virulence pathways, the Pathogen-Host Interaction (PHI) database was used to search for orthologous proteins in the *M. anisopliae* E6 genome. Comparisons with the predicted proteome of *M. anisopliae* E6 and the PHI-database were conducted employing Blastp analysis and the results were filtered according a stringent criteria (coverage ≥ 50% and e-value ≤ 10^-5^). Of the 10,817 protein-coding genes, 2,396 (22.1%) exhibited matches with proteins from the PHI database. Similar percentages of *M. robertsii* and *M. acridum* proteins also exhibited matches with the PHI databases (21.2% and 21.8%, respectively). However, *B. bassiana* had a lower percentage of PHI database matches (16.9%). Considering only proteins that show over 70% identity with *M. anisopliae* orthologs, 50 of 94 matches exhibited a “loss of pathogenicity or reduced virulence” as phenotype characteristics in mutant strains. Chitinase Chi2 (*MANI02801*, ChimaB1 in *M. anisopliae* E6) is among the classes of pathogenic proteins represented in *M. anisopliae* for which mutants exhibited reduced virulence against the cotton bug *Dysdercus peruvianus*[23]. The *M. anisopliae* protein *MANI18860* (ChimaD1 in *M. anisopliae* E6) was also found to be a putative virulence factor because this protein is homologous to the chitinase BbCHIT1 from *B. bassiana* (*AAN41259*). The overexpression of this gene in *B. bassiana* led to enhanced virulence against the aphid *Myzus persicae*[24]. Additionally, three chitin synthase coding genes could be identified (loci *MANI15599*, *MANI17339*, and *MANI112231*). *M. anisopliae* has an orthologous protein (*MANI23390*) to *M. robertsii* histidine kinase 1 (mhk1), whose null mutants showed reduced virulence to *Tenebrio molitor* larvae [25]. Superoxide dismutase (SOD), mitogen-activated protein kinases (MAP kinases), urease, Cytochrome P450 monooxygenase, and others were also proteins from *M. anisopliae* with matches in the PHI database (Additional file 1). Therefore, such proteins represent putative virulence determinants and should be exploited in future loss of function mutant experiments.

### Comparative secretome analyses

We predicted the refined secretome of *M. anisopliae* E6, and from other 15 fungal genomes available, selected from different lifestyles, such as plant pathogens (*Fusarium graminearum, Fusarium oxysporum, Magnaporthe oryzae* and *Nectria haematococca*), human pathogens (*Aspergillus fumigatus* and *Aspergillus niger*), entomopathogens (*Beauveria bassiana*, *C. militaris, M. robertsii* and *M. acridum*)*,* and mycopathogens (*Trichoderma atroviride* and *Trichoderma virens*), as well as saprophytes (*Aspergillus nidulans*, *Neurospora crassa* and *Trichoderma reesei*). The goal was to compare the secretome functionalities and to search for evolutionary traits. To perform this task, we combined bioinformatic tools (Additional file 2) based on the approach used for the plant pathogen *Fusarium graminearum*[26], which has shown high transcriptional and proteomic support. This procedure aimed to detect protein sequences encompassing signal peptides (as detected by SignalP and TargetP tools), a lack of or at most one transmembrane domain (TM) if located within the first 60 amino acids at the N-terminus (as detected by TMHMM), and sequences associated with the extracellular face of the plasma membrane via glycosylphosphatidylinositol (as detected by GPI anchors) after a post-translational modification (PredGPI). Additional cellular localization tools (ProtComp and WoLF PSort) were applied to refine the secretome predictions. Sequences lacking an initial methionine or that were smaller than 20 amino acids were excluded. To ensure that sequences known to permanently reside inside the lumen of the endoplasmic reticulum were not present, we scanned for the PROSITE pattern PS00014 (Endoplasmic reticulum targeting sequence). Our analysis relied on the association of different software modalities to improve our prediction specificity because the utilization of single programs would result in more annotation errors. For example, of the sixteen fungal species analyzed, 2.5% of the proteins predicted to have signal peptides by SignalP were not considered secreted proteins by TargetP, whereas 39.7% of the proteins predicted to have extracellular localization by WoLF PSort were rejected by ProtComp. Recent reports have revealed that the non-classical export of proteins to the extracellular space through vesicles is a conserved mechanism in fungi [27–32]. Although we are aware that the secretory pathway analyzed in this study does not represent the entire repertoire of fungal secreted proteins, notably, the classical mechanism of protein secretion is an important and well-studied route. The *M. anisopliae* E6 refined secretome represented 3.8% of the complete proteome. Similar proportions were found for all species, ranging from 3.1% (*M. acridum* and *C. militaris*) to 4.8% (*M. oryzae*) (Additional file 3). This proportion was much higher in the previously predicted *M. robertsii* secretome (17.6%); however, this number was based solely on the presence of signal peptides [18], suggesting that its secretory repertoire may have been overestimated. In contrast, our method predicted that the *M. robertsii* secretome accounts for 3.7% of the complete proteome (Additional file 3).

To evaluate the functional diversity of the secretomes studied, we employed classification based on the KEGG Orthology (KO) database and association of activities with different fungal lifestyles. The amount of predicted secreted sequences associated with functional groups varied from 33.1% (*M. oryzae*) to 62.6% (*A. niger*), indicating a considerable number of proteins with unknown functions for all fungi genomes. For example, 72% of *M. anisopliae* E6 sequences without KO functions were hypothetical proteins. Among the entomopathogens, we found that *M. anisopliae* E6 presented a higher number of glycoside hydrolase (GH) sequences, which contain both canonical signatures to be secreted and distributed to cellular compartments. GHs (EC 3.2.1.-) are ubiquitous enzymes found in all domains of life. These proteins can be both intra- and extracellular and play fundamental roles in nutrition by degrading a variety of polymeric carbohydrates [33]. Of note, when compared with fungi with different lifestyles, entomopathogens have relatively fewer GHs, secreted or non-secreted. In fungi, one important class of GH enzymes is the chitinases (EC 3.2.1.14). These proteins are classified into the GH 18 family and are assumed to be involved in insect cuticle degradation and fungi cell wall digestion during morphological changes [9, 34]. As expected, our analysis revealed that entomopathogens and mycoparasites displayed a larger set of secreted chitinases. This finding is consistent with a previous report that characterized the presence of distinct GH members in fungal genomes [35]. Although chitin is absent in mammals and in plants, human pathogens and phytopathogens present a modest set of secreted chitinases that may have antifungal roles [36]. In addition, a consistent number of non-secreted chitinases was found most likely because these chitinases have other roles in the fungal life cycle or are able to reach the extracellular space through different secretion mechanisms or by vesicle transport [28].

Comparative profiling of secretome by classification of Gene Ontology Terms was also applied in order to obtain functionalities predominant in entomopathogen secretomes in relationship to other fungal lifestyles. The annotations related to proteolysis (GO 0006508) and related (peptidase activity – GO 0008233; serine-type peptidase activity – GO 0008236) could be found as overrepresented in entomopathogens when compared to human and plant pathogens, mycopathogens as well to saprophytes (Additional file 4). To establish successful infection, entomopathogens secrete a variety of hydrolytic enzymes, such as proteases (EC 3.4.-.-). A higher number of secreted proteases in this lifestyle group was found; in *M. anisopliae* E6 and in *M. robertsii*, these secreted proteases were originated from gene family expansion of serine (EC 3.4.21.-) and aspartic endopeptidases (EC 3.4.23.-). Although it has more genes coding for trypsin (27), the *M. robertsii* genome codes for slightly fewer secreted enzymes of this class (9) than the *M. anisopliae* E6 genome (10 of 17). Considering the two types of hydrolyzing peptide bonds, the exopeptidase (EC 3.4.11.-/EC 3.4.16.-/EC 3.4.17.-) and the endopeptidase (EC 3.4.21.-/EC 3.4.23.-/EC 3.4.24.-) families are widely distributed in all analyzed secretomes.

Comparing the predicted secretomes of the three *Metarhizium* species herein analyzed, the acridid-specific *M. acridum* and the broad host-range *M. anisopliae* E6 and *M. roberstii,* fewer genes in almost all enzyme categories analyzed were found in *M. acridum* (Additional file 3). This finding is consistent with its narrow range of susceptible hosts. In agreement with this assumption, comparative genome hybridization assays [37] and a comparative genome analysis [18] revealed the absence of several genes in *M. acridum* compared with *M. robertsii*. When the secretomes of entomopathogenic fungi were compared with those secretomes from human fungal pathogens, *A. niger* was found to have the largest repertoire of proteins with a predicted PFAM domain.

**Figure 2:**
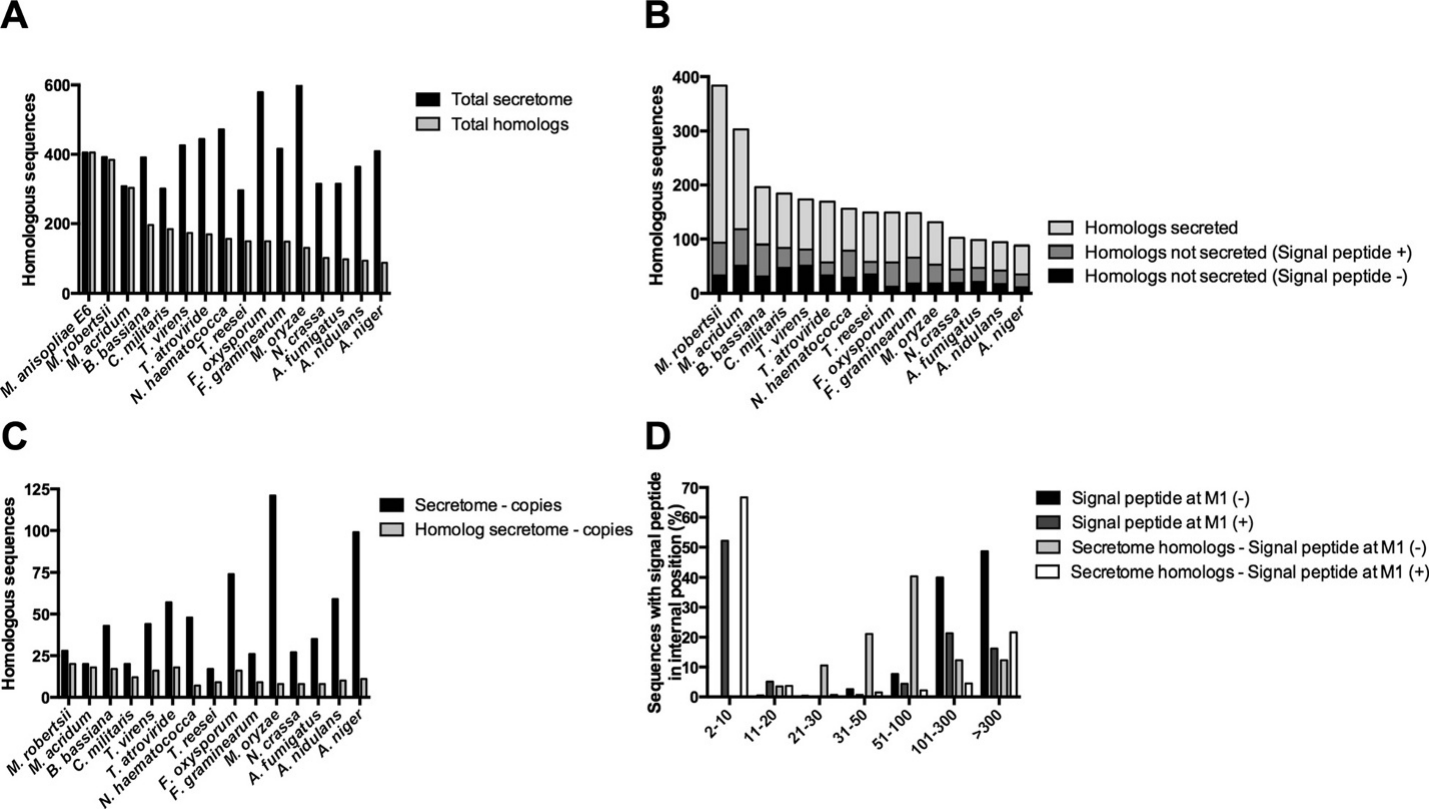
**Conservation of secreted proteins in fungi. A**. Comparison of the *M. anisopliae* E6 secretome and its homologs in fifteen analyzed fungi genomes. **B**. Prediction of signal peptide in *M. anisopliae* E6 homologs. **C**. Comparison of copy numbers of the *M. anisopliae* E6 secretome homologs **D**. Signal peptide presence in putative alternative downstream translation initiation sites for methionine.

We reasoned that fungal secretomes could share a common evolutionary trait. To evaluate this hypothesis, we performed a comparison of the predicted secretome employing the 16 genomes previously analyzed. This hypothesis was shown to be inconsistent. A reasonable explanation is obtained from comparisons of homologs present in indifferent secretomes for all 405 *M. anisopliae* E6 secreted sequences (Figure 2A and B), as well the analysis of gene duplication (≥2 copies) numbers (Figure 2C) for each predicted secretome. The number of homologs is reduced from 384 for *M. robertsii* to 88 for *A. niger*, and the duplication rates varied from 5.7% for *T. reesei* to 24.2% for *A. niger*. The closest secretomes to *M. anisopliae* E6 were the entomopathogens *Metarhizium* spp*.*, *B. bassiana* and *C. militaris*, whereas the human pathogens *Aspergillus* spp*.* was the least similar. The plant pathogen *Nectria haematococca* presented slightly more homologs (156) than the saprophyte *T. reesei* (149), whereas the other *Trichoderma* spp*.*, *T. virens* and *T. atroviride*, presented 173 and 169 homologs, respectively. These latter numbers are consistent with the reduced repertoire of *T. reesei* secreted protein families [38], including cellulases, hemicellulases and polysaccharide degradation enzymes.

The inspection of predicted secreted proteins among homolog sequences reveals that any comparative analysis based on simple homology inferences [26] should be conducted with caution. We observed that the speciation process led to differentiation in the orthologous sequences because some direct homologs between two different species differ in the presence of canonical sequences that classify a protein as secreted (Figure 2B, dark gray and black bars). *N. haematococca* showed the smallest percentage of secreted homologs (49.4%), whereas even the most taxonomically related *M. robertsii* had a much larger percentage of secreted homologs (75.8%). Considering only secreted homologs (Figure 2A, light gray bars), the mycoparasite *T. atroviride* exhibits more homologs (112) than the entomopathogens *B. bassiana* (106) and *C. militaris* (100)*.* A considerable number of homologs, ranging from 8.1% to 29.5% for *F. oxysporum* and for *T. virens*, respectively, had no detectable signal peptide (Figure 2B, black bars). This finding could represent differences between the N-terminal sequences or alternative start codon predictions [39, 40]. Additionally, the amplitude between the total predicted secretome (Figure 2A), the number of secreted proteins with coding-genes presented in copies (Figure 2C), and the total number of homologs reveals an important set of species-specific secreted proteins for each proteome compared with *M. anisopliae* E6.

The presence of alternative in-frame translation initiation sites (TIS) is a common feature that has been experimentally verified in a wide range of organisms [41–44] and that may directly influence the cellular localization of proteins [44]. Alternative TIS can occur at distances of hundreds of base pairs from the primary start codon site and has been recently predicted to be a phenomenon that occurs in approximately one-tenth of all *Saccharomyces cerevisiae* proteins [42]. In an attempt to detect signal peptides in a downstream methionine, which could be an alternative TIS, the *M. anisopliae* E6 predicted proteome and its homolog sequences were split, beginning with all methionines present at the second to the last 30 residues to the end of each protein. Subsequences were screened for the presence of signal peptides by both SignalP and TargetP tools and were subsequently divided into two categories: (i) signal peptides already detected at the first methionine (M1) of the primary sequence and (ii) signal peptides not detected (Figure 2D). From the 10,817 *M. anisopliae* E6 coding sequences analyzed, 9,388 could be split into subsequences. Of these subsequences, 987 had signal peptides detected at M1 (category (i)). For 112 (11.3%) of these 987 proteins, an additional signal peptide could be detected (Figure 2D, dark gray bars), accounting for 136 subsequences. Approximately 57.3% of these methionines were located within 20 residues of M1, indicating that M1 may be the same motif detected, although 37.5% were located up to 100 amino acid residues from M1. For 886 (10.5%) of the 8,401 proteins from category ii, a signal peptide motif could be identified in a downstream methionine (Figure 2B, black bars), accounting for 1,254 subsequences. In 88.7% of these sequences, an alternative TIS was present over 100 residues downstream of M1. The same analysis was applied to any proteins classified as secretome-related. Of the 2,524 *M. anisopliae* E6 secretome homologs, 2,056 could be split into subsequences. Then, these sequences could be grouped into categories (i), with 1,787 sequences, and (ii), with 269 sequences. One hundred and twenty-eight sequences (7.2%) of the group with 1,787 proteins (category (i)) showed an additional motif (alternative TIS), resulting in 134 subsequences (Figure 2B, white bars), of which 70.1% most likely accounted for the same signal within 30 residues of M1. Finally, for only 52 (19.3%) of the 269 proteins (category (ii)), a signal peptide was detected downstream of M1 (Figure 2B, light gray bars), adding 57 subsequences to the alternative TIS, with 35.1% within 50 residues from M1. Together, these results indicate that even if alternative TIS is actually occurring in a downstream methionine for these proteins, the majority of homologs without a detectable signal peptide at M1 may not be secreted, similar to *M. anisopliae* E6. Again, the possibility of alternative secretion mechanisms cannot be excluded. In fact, these results strongly suggest that such mechanisms exist and may account for the secretion of an important number of proteins.

Among the predicted extracellular proteins of *M. anisopliae* E6, we could identify 80 glycosylphosphatidylinositol-anchored proteins (GPI-Ps). This post-translational modification has been implicated in protein sorting, trafficking and dynamics in different cells [45]. In yeast, GPI lipids are synthesized in the endoplasmic reticulum, and their addition to target proteins is conducted by a pathway that is composed of 12 steps [46]. The number of GPI-Ps in *M. anisopliae* E6 is higher than that in other *Metarhizium* spp. (68 for *M. robertsii* and 63 for *M. acridum*) and lower than that in the two other entomopathogens (73 for *C. militaris* and 76 for *B. bassiana*). The majority (60%) of the GPI-Ps identified represent conserved hypothetical proteins. However, the GPI-Ps of *M. anisopliae* E6 showed considerable functional diversity, as revealed by the analysis of conserved domains (Additional file 5). Of the 80 predicted GPI-Ps, 11 proteins could be classified as glycoside hydrolases, whose orthologs were characterized in *N. crassa*[47], in *A nidulans*[48], as well in *M. robertsii*[49].

To identify possible GPI-P orthologs shared by the genome sequences of *Metarhizium* spp., we conducted a BLAST analysis for *M. anisopliae* E6, *M. robertsii* and *M. acridum.* We found that most of the *M. anisopliae* E6 GPI-Ps had orthologs in both *M. robertsii* and *M. acridum* that also had a GPI anchoring signal. However, in some cases, the GPI-Ps from *M. anisopliae* had orthologs only in either *M. robertsii* or *M. acridum*, or even had exclusive proteins (Additional file 6). In addition, despite the clear presence of orthologs among the three *Metarhizium* spp., a few orthologous proteins to *M. anisopliae* E6 GPI-Ps differed only in the presence or absence of the GPI anchoring signal in the *Metarhizium* spp*.* counterparts, suggesting a possible difference in their cellular location (Additional file 6). These data suggest that there are differences in their protein cell surface profile, despite their phylogenetic proximity. Indeed, the differences observed in their GPI-P profiles may represent differences in fungal survival in the environment, virulence and host specificity.

Because secretory proteins play a fundamental role in fungi physiology and because their evolution is essential for fungal fitness, gene duplication rates could be significantly higher within secretome genes when compared with the remnant proteome. For the fungal pathogen *F. graminearum*, genes coding for secreted proteins have been preferentially found in chromosomal regions with higher recombination frequencies [26]. Additionally, gene duplication is an important source of new biological functions because mutations in one of the copies can affect protein structure without being deleterious, whereas the other copy can retain functionality [50, 51]. To evaluate the prediction that secretome coding genes are more susceptible to duplication than the proteome coding genes as a whole, a proportion test was conducted. This analysis supported this hypothesis for the two human pathogens *A. fumigatus* (p-value < 0.001) and *A. niger* (p-value < 0.001), for the plant pathogen *M. oryzae* (p-value < 0.001), for the three saprophytes *A. nidulans* (p-value < 0.001), *N. crassa* (p-value = 0.002) and *T. reesei* (p-value = 0.046) and for the two mycoparasites *T. atroviride* (p-value < 0.001) and *T. virens* (p-value = 0.002) (Additional file 7). Conversely, for *N. haematococca* (p-value = 0.024), this proportion was inverted, such that secreted protein coding genes had less duplication. This finding was consistent with the percentage found for predicted secreted proteins and with the higher duplication rate in supernumerary chromosomes [52]. *M. anisopliae* is adapted to a diverse range of niches [18, 53, 54], and our analysis revealed that the proportions of duplications for the secretome coding genes compared with the proteome coding genes as a whole are statistically equal. This finding suggests that for entomopathogenic fungi, in contrast to human and plant pathogens, successful adaptation to different habitats may be more qualitative. This hypothesis argues that the presence or absence of specific genes, in contrast to gene duplications, is required for the adaptation of *Metarhizium* spp. to different habitats.

**Figure 3:**
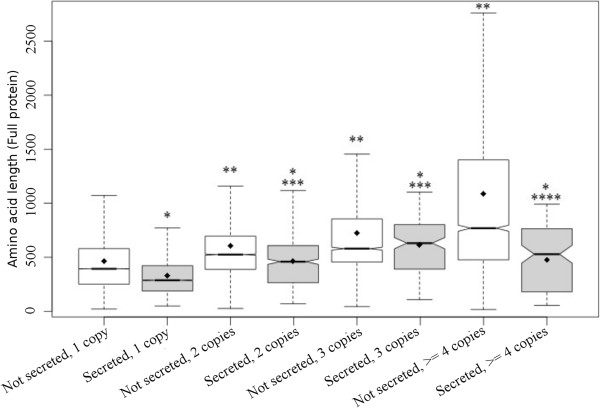
**Gene duplication and protein size box-plot for all sequences predicted to be secreted or non-secreted.** Mean protein sizes, represented as black dots, were compared with a one-tailed Student’s t-test. *Secreted mean smaller than the non-secreted mean with same copy number, **Non-secreted mean greater than one copy less than the non-secreted mean, ***Secreted mean greater than one copy less than the secreted mean, ****Secreted mean smaller than one copy of the less secreted mean. All p-values < 0.001. Four or more copies were grouped together.

Longer (more complex) duplicated proteins are more likely to be retained because these proteins have a higher probability of generating new biological functions, as previously observed for several fungal species, including saprophytes and human pathogens [51]. Considering the mean protein size for all sixteen analyzed fungi species, we found that duplicated genes are larger than single copy genes for both the secreted and non-secreted sequences (Figure 3) and that longer genes have more copies (with the exception of secreted proteins with four or more copies). Additionally, independent of the copy number, secreted proteins are generally smaller than the average size of the rest of the proteome (All p-values < 0.001 on one-tailed Student’s t-test). Therefore, evolutionary mechanisms for the selection of secreted proteins based on their size must have occurred to allow for their translocation into the extracellular space or to allow for those proteins to function properly in that external environment.

### Transcriptome analysis

**Figure 4:**
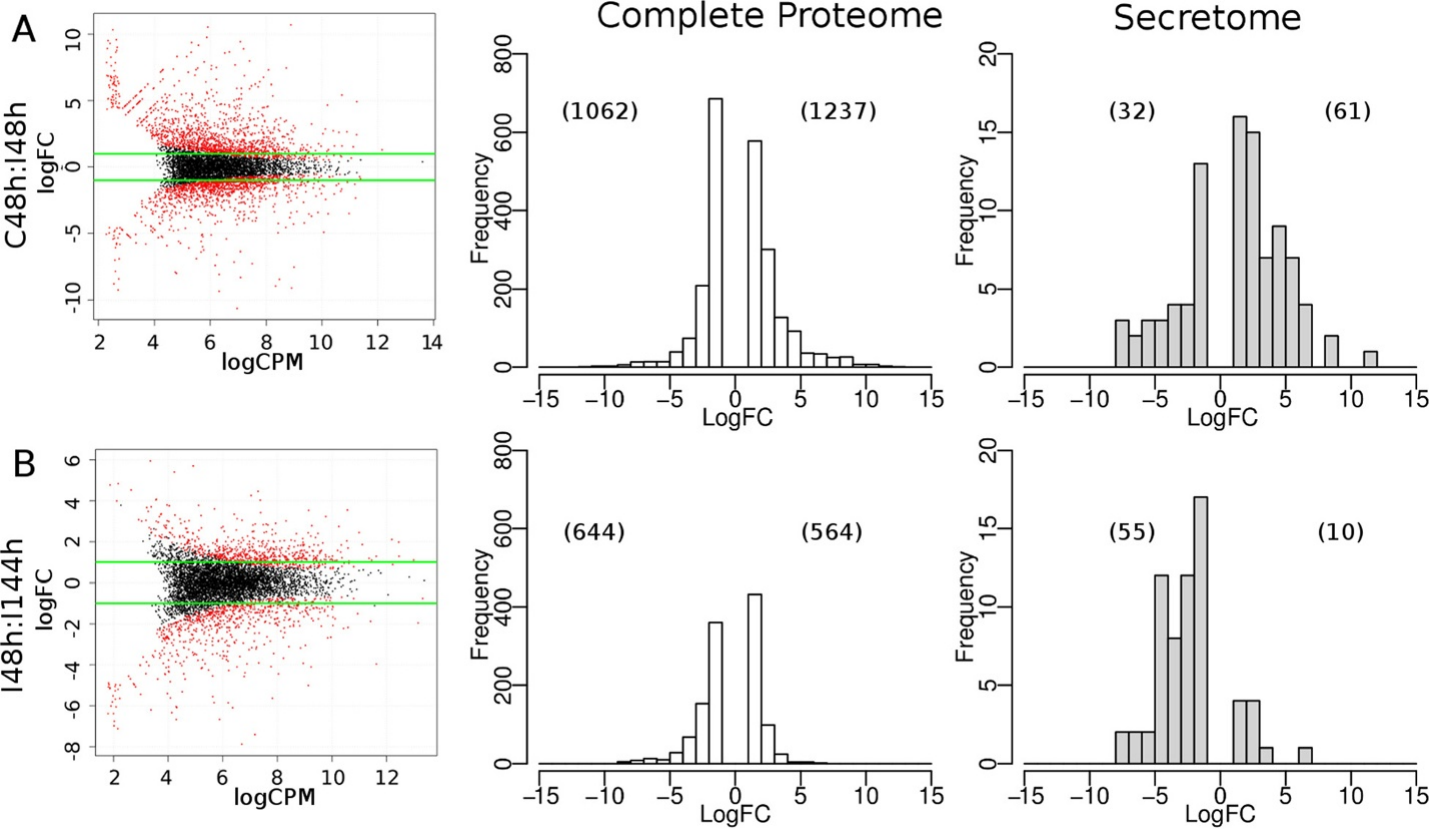
**RNA-Seq analysis of differentially expressed genes from** ***M.*** ***anisopliae*** **E6.** Comparison of the expression profile from C-48 h (Control-48 h) and I-48 h (Infection-48 h) **(A)** or I-48 h (Infection-48 h) and I-144 h (Infection-144 h) **(B)** was conducted with the edgeR software package. The overall expression of genes (left panel), the number of differentially expressed genes (middle panel), and the secretome-associated genes (right panel) are shown.

We conducted the first RNA-Seq experiment to evaluate the differential gene expression profiles of *M. anisopliae* E6 acting on tick (*Rhipicephalus microplus*) cuticles to mimic the infection process (as described in the Methods section). These experiments were conducted in three different conditions: (i) a control condition, with spore suspensions cultured in complete medium for 48 hours (C-48 h); (ii) an early cuticle infection condition (I-48 h), with tick cuticles inoculated with spore suspensions and incubated for 48 h in water-agar plates; and (iii) a late cuticle infection condition (I-144 h), with tick cuticles inoculated with spore suspensions and incubated for 144 h in water-agar plates. After mapping reads from all three experimental setups to the genome, approximately 89% of the 10,817 predicted protein coding sequences had at least two uniquely mapped reads (Additional file 8). Notably, the transcriptome results validated our genome annotation since reads that mapped to 415 of the 690 *M. anisopliae* exclusive genes (60%) could be detected (Additional file 8). The statistical package edgeR [55], which has recently shown better performance considering speed and accuracy than other frequently used tools [56], was used in two pairwise comparisons between the RNA samples, revealing distinct patterns of gene regulation in the tree experimental setups. A principal component analysis (PCA) showed a satisfactory degree of variability among the biological replicates (Additional file 9). The condition mimicking early infection (C-48 h × I-48 h, Figure 4A) triggered a higher amount of up-regulated genes (1,237 genes (FDR ≤ 0.05, log_2_FC ≥ 1)) compared with down-regulated genes (1,062 genes (FDR ≤ 0.05, log_2_FC ≥ 1)), with similar proportions of predicted secreted sequences (4.9% and 3.0%, respectively). After an additional 4 days of fungal contact with the host cuticle (I-48 h × I-144 h, Figure 4B), the scenario was inverted, with more genes down-regulated (644) than up-regulated (564), whereas the proportion of the predicted secretome was 8.5% and 1.8%, respectively. Considering secretome genes with more than two mapped reads in any replicate, 55.6%, 59.5% and 47.4% were expressed for setups C-48 h, I-48 h and I-144 h, suggesting that most of these proteins may be constitutively expressed in the condition analyzed in this study.

**Figure 5:**
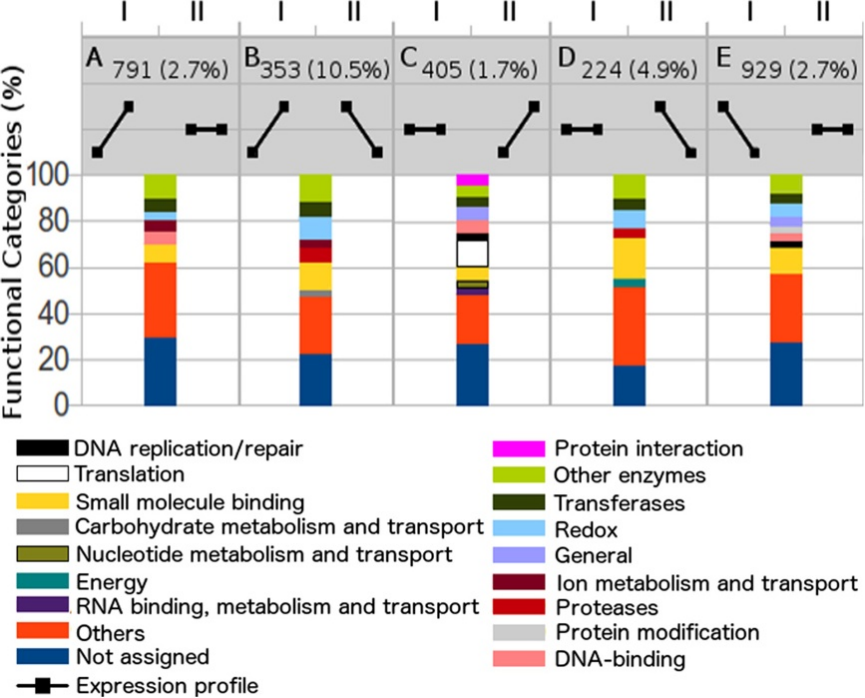
**Superfamily functional categories of identified expressed genes.** The total gene numbers and the percentages of the predicted secretome are for each of the five distinct regulation profiles **(A-E)**. I: edgeR pairwise comparison C-48 h:I-48 h; II: I-48 h:I-144 h. F: Nucleotide m-tr*; G: Carbohydrate m-tr*; HA: Small molecule binding; J: Translation; LA: DNA-binding; O: Protein modification; OA: Proteases; OB: Kinases-phosphatases; P: Ion m-tr*; Q: Secondary metabolism; R: General; RA: Redox; RB: Transferases; RC: Other enzymes. *m-tr: Metabolism and transport. Categories with representativeness < 3% were grouped as Others. The complete classification can be found at: http://supfam.cs.bris.ac.uk/SUPERFAMILY/function.html.

To observe the possible biological roles of differentially expressed genes, superfamily functional categorization [57] was applied (Figure 5). At least 5 different expression profiles could be detected, which encompassed the expression variation of 2,702 genes. This classification is based on the differential expression of functional categories when comparisons between C-48 h and I-48 h (Panels I) and I-48 h and I-144 h (Panels II) were conducted (Figure 5, A to E). The majority of the differentially expressed genes fell into category A (up-regulated in the infection condition compared with the control condition) or E (down-regulated in the infection condition compared with the control condition). Neither the A or E profiles showed differences in their gene expression between the I-48 h and I-144 h conditions; however, these profiles contain differences when C-48 h and I-48 h are compared. In all profiles, genes under constant regulation from the categories “HA: Small molecule binding” (e.g., EC 1.1.1.-/EC 1.3.1.-, binding to NAD or NADP; EC 1.14.13.-, binding to NADH or NADPH; and EC 1.5.3.-, binding to oxygen), “RB: Transferases” (e.g., EC 2.1.1.-, methyltransferases; EC 2.6.1.-, transaminases) and “RC: Other enzymes” were identified. The “RA: Redox” category was also observed in almost all profiles (e.g., EC 1.14.-.-, incorporation or reduction of molecular oxygen). As described for other *Metarhizium* species [18], the cAMP response element-binding (CREB) protein, which is a major downstream transcription factor in mammals that has not yet been characterized in fungi, was up-regulated (Figure 5A and Additional file 10). A considerable proportion of “G: carbohydrate metabolism and transport” and “OA: protease” categories in profile B is of note because these categories represent gene products involved in the early stages of infection (profile B, up-regulated in I-48 h compared with C-48 h; Figure 5B). Moreover, profile B harbored the highest set of genes (10.5%/37 genes) coding for proteins predicted to be secreted, which is consistent with the expected function of the secretome (Additional file 10). Four subtilisins, which are important enzymes for host cuticle degradation and nutrition [58], were detected in this expression profile (Additional file 10), along with proteins CAS1 and MAS1, whose orthologs in plant pathogens are known to be involved in appressorium formation [59]. Continued contact with the cuticle after 144 hours enhanced the expression of categories “A: RNA binding, metabolism and transport”, “F: Nucleotide metabolism and transport”, “J: Translation” and “RD: Protein interaction” (Figure 5C, profile C, Figure 5C). tRNA synthetases, which are the enzymes responsible for charging the correct amino acid to its cognate tRNA, as well as enzymes acting on ribosome biogenesis and cell cycle progression, were up-regulated [60], indicating that the fungal cells were metabolically active (Additional file 10). As the host cuticle is exhausted, proteases are down-regulated (Figure 5B and D, profiles B and D), and primary metabolism is reduced. Thus, the category “C: energy”, which includes glycolysis and tricarboxylic acid cycle components (e.g., EC 1.2.1.12, Glyceraldehyde 3-phosphate dehydrogenase; EC 5.3.1.1, Triosephosphate isomerase and EC 2.3.3.8, ATP citrate synthase (Additional file 10), was down-regulated, as shown in profile D (Figure 5D).

In this study, two trypsin (EC 3.4.21.4) isoforms, which are a class of serine proteases, were found to be up-regulated during early infection, consistent with previous findings [61]. However, one isoform was highly down-regulated (Additional file 10), which could represent differential ambient pH responsiveness [62]. All three isoforms were predicted to be secretome components. Although trypsins are usually more active at an alkaline pH, aspartic endopeptidases (EC 3.4.23.-) are more active at an acidic pH. Five predicted secreted aspartic endopeptidases presented differential expression levels with distinct responses to the environmental conditions. Two were highly down-regulated, whereas the other three were up-regulated during early or late infection (Additional file 10). Additionally, chymotrypsins, which are another class of proteases that were likely originated from horizontal gene transfer from bacteria [63], were also present in two copies in *M. anisopliae* E6 (*MANI06361* and *MANI115263*, orthologs to *M. robertsii MAA_07484*). The RNA-Seq analysis revealed that these genes did not present differential transcript levels in the conditions evaluated in this study. Although these enzymes are important for tick cuticle chitin degradation and for fungi cell wall remodeling, no chitinase was up-regulated in the conditions analyzed in this study. One chitin synthase (EC 2.4.1.16) was up-regulated.

The gene that was most down-regulated during the contact period with the host cuticle was a nitrate reductase (EC 1.7.1.3), which is an enzyme that is essential for reducing nitrate to ammonia. This down-regulation can be explained by the physiological condition of nitrogen starvation faced by the fungal cells, which have been shown to be essential for activating distinct virulence functions in plant pathogenic fungi [64]. In accordance, starvation-stress gene A (ssgA), which is a hydrophobin-like protein that leads to decreased fungal sporulation and virulence when deleted [65], was highly up-regulated (Additional file 10).

Infection metabolism induction may produce derivatives of reactive oxygen species (ROS), which are capable of causing damage to diverse cell components, as byproducts. Examples of ROS molecules include hydrogen peroxide (H_2_O_2_), superoxide anions (O_2_^-^) and nitric oxide (NO). As a defense mechanism against this oxidative stress, superoxide dismutase (EC 1.15.1.1) expression was up-regulated for the conversion of superoxide into O_2_ and H_2_O_2_. Similarly, catalase (EC 1.11.1.6) and catalase-peroxidase (EC 1.11.1.21) expression were up-regulated for H_2_O_2_ inactivation, and glutathione S-transferase (EC 2.5.1.18) expression was up-regulated for neutralizing electrophilic substrates. Additionally, a thioredoxin reductase (EC 1.8.1.9) isoform responsible for reducing thioredoxins, which can act as antioxidants by reducing other proteins, was down-regulated. The accumulation of thioredoxin in its oxidized form may also have a protective role because thioredoxin is an effective cysteine oxidant [66] that regulates protein disulfide bond formation in the reducing environment of the cytoplasm, resulting in the downstream regulation of oxidative stress transcriptional factors and chaperones [66]. Peroxiredoxin, which is also an antioxidant enzyme capable of degrading H_2_O_2_ (EC 1.11.1.15), was also down-regulated because this enzyme requires the scarce reduced thioredoxin form for its proper function (Additional file 10). For completeness, edgeR statistical analyses are provided (Additional file 11).

### Phylogenomic analyses

**Figure 6:**
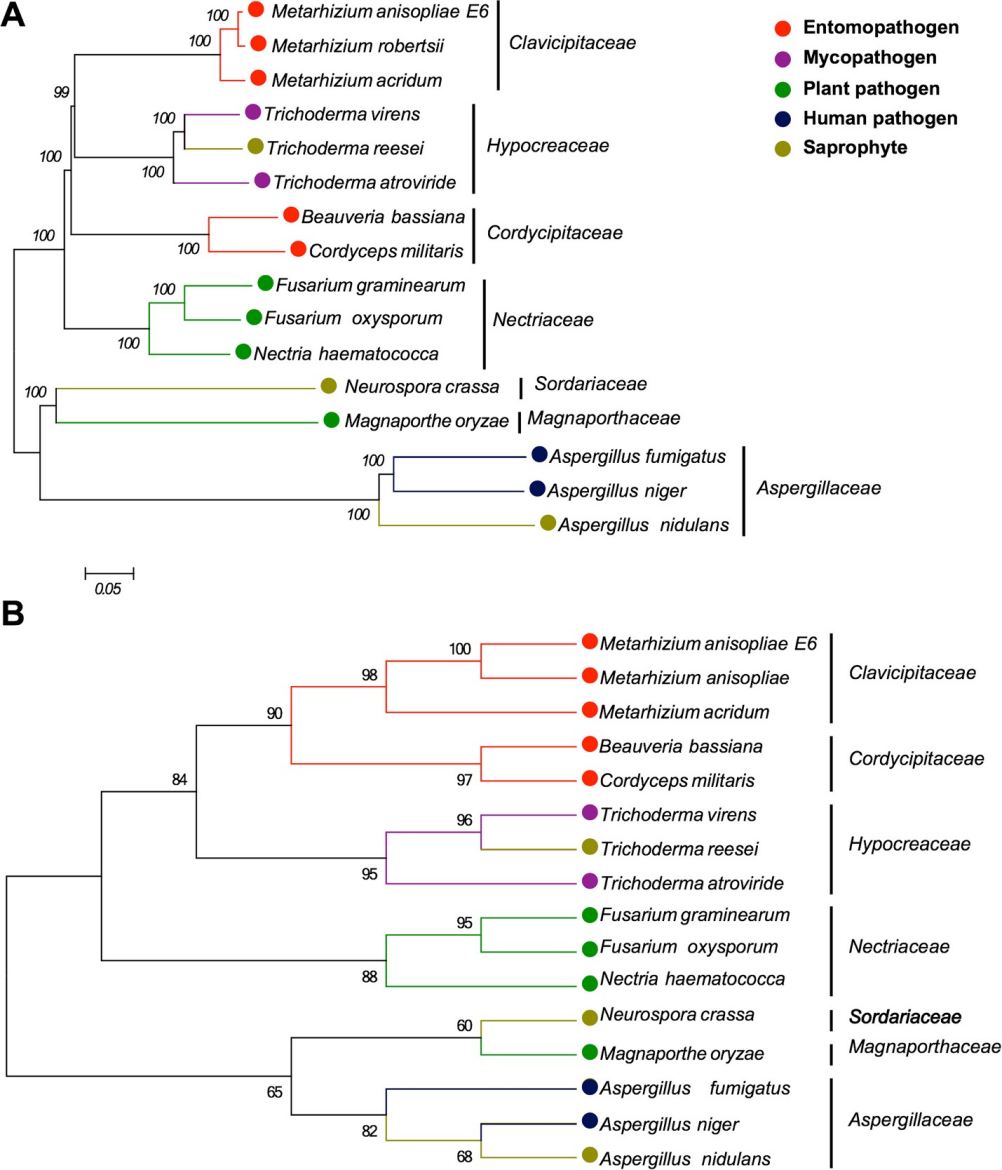
**Fungal evolutionary history obtained by using a phylogenomic approach.** NJ methods were employed to all orthologs identified in the fungal genomes **(A)**. Nearest neighbor interchange method with a neighbor-joining (NJ) tree calculated from average consensus distances were employed to all orthologs identified in the fungal secretomes **(B)**. The percentage of replicate trees in which the associated taxa clustered together in the bootstrap test (1,000 replicates for genomic and 100 for secretomic analysis) is shown next to the branches. The scale bar represents substitutions of amino acids per site.

As the content of the predicted secretomes change according to the fungal species analyzed, homology relations were established among *M. anisopliae* E6 and a set of fifteen previously cited fungi to evaluate and to compare the evolutionary patterns of the *M. anisopliae* genome and secretome. In addition a correlation with the transcriptomic data was conducted. All 1:1 ortholog sequences representing different CDSs were concatenated, leading to an aligned file containing 1,947,162 amino acid residues. As expected, the *Metarhizium*, *Trichoderma*, *Fusarium*, and *Aspergillus* genera formed monophyletic clusters [38, 67–70]. The genera *Neurospora* and *Aspergillus* clustered together, with high bootstrap support. Additionally, *Metarhizium* and *Trichoderma* formed a statistically well-supported clade in the *supermatrix* and *supertree* approaches applied to the shared proteome, which was formed by orthologous proteins only (Figure 6A). When considering the major clades obtained using the maximum likelihood (Figure 6A) and the distance phylogenetic methods (Additional file 12), the trees architectures did not changed significantly.

Some fungal genera, including *Trichoderma* and *Metarhizium*, evolved a variety of nutrition strategies. For instance, some *Trichoderma* species are mycotrophic and, therefore, can grow on living fungi in a process known as mycoparasitism, whereas other species are saprotrophic [71]. *Metarhizium* species have already been isolated from diverse habitats, primarily from dead arthropod carcasses but also from mycorrhiza and as endophytic [72]. The shared proteome also indicates that the plant pathogens *F. graminearum*, *F. oxysporum* and *N. haematococca*[52, 68, 73] are related to the *Metarhizium* clade. Wyrebek and coworkers [54] have proposed that *Metarhizium* species show plant-specific rhizosphere associations within a habitat. Moreover, *M. robertsii* was also shown to be an endophyte that stimulates root development [53], thus making the direct transfer of nutrients from fungal to plant cells possible [74–76]. Thus, the phylogenomic analysis presented in this study reveals that determining the life-style of *M. anisopliae* is far more complex than the global set of genes can predict.

The phylogenomic analysis also indicated that *M. anisopliae* E6 and *M. anisopliae* ARSEF 23, now identified as *M. robertsii*[18], share a high number of orthologs. These species formed a statistically well-supported clade, with *M. acridum* basal to these species, corroborating the results of Bischoff and coworkers [17]. Notably, *M. robertsii* is morphologically indistinguishable from *M. anisopliae.* Therefore, phylogenetic analysis is critical for establishing the identity of these fungi.

A phylogenetic analysis was conducted for 212 ortholog-groups representing all proteins that had been identified as putative constituents of the secretome. The evolution of the secretome indicated an important difference when compared with the evolutionary history of these fungi based on their shared proteome. There is a closer evolutionary relation between the *Metarhizium* clade and that formed by the genera *Beauveria* and *Cordyceps* (Figure 6B). Consequently, most proteins secreted by *Metarhizium* species are similar to those proteins from the *Beauveria* and *Cordyceps* genera. These proteins, some of which are important to the infection process, have a pattern of evolution that is extremely similar in fungi with similar hosts.

To identify the proteins responsible for the distinctive evolutionary pattern of the secretome, all individual phylogenies had their topologies compared with the topology of the phylogenomic tree. At least one *Metarhizium* and one *Trichoderma* sequence, as well as one *Cordyceps* or *Beauveria* sequence, had to be present in a specific secretome ortholog file to be included in the analysis. Finally, 37 secreted proteins were identified as being potentially responsible for the different pattern of evolution of the secretome when compared with the evolution of the organisms represented by the phylogenomic tree. These proteins may explain why fungi with similar hosts cluster together. Thirty four (92%) of those genes are expressed, and 16 (43%) of those genes are differentially expressed during early cuticle infection (I-48 h) and/or during late cuticle infection (I-144 h) (Additional file 13). The biological significance of these findings requires further investigation. Nevertheless, our results show that specific secreted proteins from *Metarhizium*, *Beauveria* and *Cordyceps* species, have an evolutionary history that points for their adaptability to host infection.

Using either proteome or secretome data, *M. oryzae* and *N. crassa* form a basal cluster with high bootstrap support. These species belong to the distinct orders Magnaporthales and Sordariales, respectively, and our results corroborate a previous study that noted their evolutionary relation [77]. The *Aspergillus* genus, which belongs to the Eurotiomycetes order, clusters with the *M. oryzae* and *N. crassa* clades and, together, represents the most basal clade in our study. The operational taxonomic units belonging to Hypocreales also form a cluster. These results indicate that the orthologs identified for the proteome and for the secretome have an evolutionary pattern that is consistent with the actual taxonomic classification of fungi.

## Conclusions

The *M. anisopliae* E6 genome and expression profiling analyses provide insights into the molecular mechanisms for adapting to the distinct lifestyles of this entomopathogenic fungus. The comparative analyses presented in this study reveal that *Metarhizium* spp. genomes harbor a complex set of genes coding for secreted proteins. Such secreted proteins appear to have been selected and maintained in the genome to cope with the distinct lifestyles presented by *Metarhizium* spp., which can be either entomopathogen-, endophytic- or rhizosphere-associated. The transcriptome profiling of *M. anisopliae* exposed to infection-mimicking conditions compared with laboratory growth conditions showed many genes that were differentially expressed. Among these genes, many genes coding for secreted proteins could be found, which could represent *M. anisopliae* virulence determinants. Our results offer selected sequences for further characterization of secreted proteins with potential roles in the *M. anisopliae* infectious process.

## Methods

### Sample collection and DNA extraction

*M. anisopliae* var. *anisopliae* strain E6 was isolated from *Deois flavopicta*[11] collected in Espírito Santo State, Brazil. This strain was incubated at 28°C for 48 h in CM (Cove’s Medium) liquid medium [78] for the subsequent DNA isolation, as previously described [23]. The genomic DNA was extracted from the mycelium cultivated in CM and was further purified using a DNeasy Plant Mini Kit (QIAGEN, Hilden, Germany). The quality of the isolated genomic DNA was assessed spectrophotometrically.

### Genome sequencing, assembly and annotation

Two shotgun (SG) and one long paired-end (LPE) libraries were constructed using approximately 5 μg of DNA each. The library construction, titration, emulsion PCR and sequencing steps were performed according to the manufacturer’s protocol without modifications. SG libraries were sequenced using GS FLX Titanium chemistry (454-Roche, Brandford, CT, USA). One of the libraries was sequenced in one region of a two-region PicoTiterPlate (PTP) and the other in both regions of a two-region PTP. The LPE library was sequenced using GS FLX standard chemistry (454-Roche, Brandford, CT, USA) in both regions of a two-region PTP.

Replicates [79] software was used to identify and eliminate the artificially replicated sequences produced during the 454-based pyrosequencing. Newbler Assembler version 2.8 and WGS-CA 7.0 software were used to perform the assembly procedures. Minimus2 software [80] was applied to obtain a consensus assembly. The remainder gaps were filled using Consed software [81].

All contig sequences were analyzed and functionally annotated using the System for Automated Bacterial Integrated Annotation (SABIA) [82] altered to annotate eukaryotic genomes by the use of AUGUSTUS [83]. The automatic annotation criteria for assigning an ORF as “valid” included ORFs with BLASTp hits on KEGG, NCBI-nr or UniProtKB/Swiss-Prot databases, respectively; subject and query coverage ≥60%; and positives ≥60%. ORFs with no BLASTp hits found on NCBI-nr, KEGG, UniProtKB/Swiss-Prot, TCDB and Interpro databases or not included in the criteria above were defined as “hypothetical” ORFs.

### Selection of refined secretomes and functional analyses

The *M. anisopliae* strain E6 predicted proteome, as well as those proteomes from fifteen other filamentous fungi (*Aspergillus fumigatus* Af293, *Aspergillus nidulans* FGSC A4, *Aspergillus niger* CBS 513.88, *Beauveria bassiana* ARSEF 2860, *Cordyceps militaris* CM01, *Fusarium graminearum* PH-1, *Fusarium oxysporum* f. sp. *cubense race 1*, *Metarhizium anisopliae* ARSEF 23, *Metarhizium acridum* CQMa 102, *Magnaporthe oryzae* 70-15, *Neurospora crassa* OR74A, *Nectria haematococca* mpVI 77-13-4, *Trichoderma atroviride* IMI 206040, *Trichoderma reesei* QM6a, and *Trichoderma virens* Gv29-8) were downloaded from the NCBI genome database (http://www.ncbi.nlm.nih.gov/genome/) and considered for *in silico* secretome analysis.

The prediction of all refined secretomes was based on the procedure described by Brown and coworkers [26] for the plant pathogen *Fusarium graminearum*. An automatic pipeline was developed using PERL scripts and the MySQL database. Initially, all proteins were screened to remove sequences without an initial methionine and with a mature peptide size of less than 20 amino acids. To detect a signal peptide, proteins with predictions by both SignalP v4.1 [84] (D-score = Y; http://www.cbs.dtu.dk/services/SignalP/) and TargetP v1.1 [85] (LOC = S; http://www.cbs.dtu.dk/services/TargetP/) tools were selected. These proteins were subsequently scanned for the presence of transmembrane regions using the hidden Markov model topology predictor TMHMM [86] (TMHMM v2.0; http://www.cbs.dtu.dk/services/TMHMM/), and we kept those proteins with 0 or 1 TM when a single TM was in the first 60 amino acids in the N-terminal portion. This filtering was necessary as the large majority of secreted proteins spam at the amino terminus of the protein at most 1 TM region, which resembles the signal peptide. PredGPI [87] (FRate ≤ 0.005) was used to predict GPI-anchors (http://gpcr2.biocomp.unibo.it/predgpi/pred.htm). ProtComp v9.1 (with LocDB and PotLocDB, proteins predicted as secreted by both NNets and Integral predictions; http://www.softberry.com) and WoLF PSort v0.2 [88] software were combined to infer the protein localization for the fungi studied (Extr ≥ 17). Finally, a PROSITE scan [89] was used to remove sequences associated with the pattern PS00014 (Endoplasmic reticulum targeting sequence), yielding the refined secretome with GPI-anchored proteins (GPI-Ps).

To assign a predicted function, a BLASTp search (e-value 1e-5) was conducted with selected proteins against the KEGG Orthology [90] database (KO). To avoid spurious domain alignments, we discarded BLAST results for which the alignment size divided by subject size (coverage) was below 50%. Those proteins without significant hits were analyzed with a PFAM-A database using pfam_scan.pl script [91]. The Pathogen-Host Interactions (PHI) database [92] (http://www.phi-base.org/) was used to search for orthologous proteins in *M. anisopliae* using an e-value of 10^-5^ and ≥ 50% coverage as criteria*.* Then, the matches were filtered, and only proteins that shared over 70% identity with *M. anisopliae* predicted proteins were included. Of these proteins, proteins exhibiting a “loss of pathogenicity or reduced virulence” as phenotype characteristics in the mutant strains were analyzed.

Statistical analyses were conducted using the R statistical package. One-tailed proportion test (prop.test) was used for evaluate secretome gene duplications. Statistical analysis for the comparison of GO enriched terms was conducted with Blast2GO [93].

### Transcriptome analysis

*Rhipicephalus microplus* cuticles were sterilized and used as the sole nutrient source for *M. anisopliae* E6 growth and development. A spore suspension (5 × 10^6^ spores per ml) was used to inoculate the cuticles by immersion for 30 sec. The inoculated cuticles were disposed over 1% water agar plates and maintained for 48 h and 144 h at 28°C. Each of the two biological replicates consisted of a pool of five plates containing mycelium growth over the cuticles. The comparative control condition was conducted on 100 ml liquid complete medium for 48 h at 28°C. The resulting fungal growth over the host cuticle and on liquid medium was ground to a powder in liquid nitrogen, and the total RNA was extracted using TRIzol® Reagent (Life Technologies, CA, USA) following the manufacturer’s instructions. The total isolated RNA was subjected to DNase treatment using RNase-free DNase I (Thermo Scientific, MA, USA). Large ribosomal RNA molecules were selectively depleted from the total RNA using a RiboMinus™ Eukaryote Kit for RNA-Seq (Life Technologies, CA, USA), and mRNA was concentrated and purified on a RiboMinus™ Concentration Module (Life Technologies, CA, USA).

RNA-Seq was conducted with Ion Torrent technology in an Ion Proton System. FastQC v0.10.0 [http://www.bioinformatics.babraham.ac.uk/projects/fastqc/] software was used for a reads quality check, and the FASTX-Toolkit v0.0.13 (http://hannonlab.cshl.edu/fastx_toolkit/) was used for trimming. Reads smaller than 30 nucleotides were discarded. The remaining reads were mapped to the *M. anisopliae* genome using the spliced read mapper Tophat v2.0.10 [94] with default parameters. HTSeq v0.5.4p5 (http://www-huber.embl.de/users/anders/HTSeq/doc/overview.html) was used to count reads aligned to only one position in protein coding regions, and the edgeR package v3.4.0 [55] was used to assess for differential gene expression, with a 5% false discovery rate (FDR ≤ 0.05) and with stringent log fold variation (logFC) ≥ or ≤ -1. Two pairwise comparisons were performed for periods C-48 h × I-48 h and I-48 h × I-144 h. InterProScan was used to assign more general superfamily functional categories v1.73 [57].

### Phylogenetic and phylogenomic analyses

OrthoMCL v2.0.8 software [95] was used with default parameters to identify orthologs and paralogs among the complete proteomes of all sixteen studied organisms. A PERL script was developed to select only 1:1 orthologous sequences from the OrthoMCL output such that only a single gene copy was selected from each predicted proteome. The multi-FASTA ortholog files of each protein sequence were used as input for the multiple alignments using CLUSTAL Omega algorithm [96] with default parameters. Subsequently, SCaFos software [97] was used to for the gene concatenation of the 2,684 alignment files. Phylogenies involving the concatenated deduced amino acid sequences from all species were evaluated through distance and probabilistic methods using the PHYLIP package [98], MEGA 5.2 Computing Core [99] and TREE-PUZZLE [100] software.

Initially, multiple 100 bootstrapped data sets were generated by the Seqboot program of the PHYLIP package. Then, these data sets were submitted to ProtDist software analysis to compute a distance matrix under the JTT (Jones-Taylor-Thornton) model of amino acid replacement. The Neighbor software applied the neighbor-joining (NJ) method [101] to the resulting multiple data sets, building trees through the successive clustering of lineages. A consensus tree was obtained using the Consense program of the PHYLIP package. Three different distance matrices (p-distance, Poisson, and JTT) were evaluated using the MEGA 5.2 Computing Core, with the complete deletion and pairwise deletion options for the treatment of gaps. The bootstrap test for phylogeny was performed using 1,000 repetitions.

The quartet-puzzling [102] search algorithm implemented by TREE-PUZZLE was used to reconstruct phylogenetic trees according to the maximum likelihood (ML) approach. The JTT model of amino acid substitution was applied. The quartet-puzzling tree topology was based on 1,000 puzzling steps. The consensus tree was constructed based on a 50% majority rule consensus. The TreeView program [103] and MEGA 5 software were used to visualize and to edit the resulting phylogenies.

In total, 212 ortholog files of the secretome were submitted to phylogenetic analysis using distance methods implemented by MEGA software. The neighbor-joining algorithm, with pairwise deletion of gaps, was applied to the set of data. The p-distance, poisson and JTT matrices were evaluated. The bootstrap test of phylogeny was performed using 1,000 repetitions.

The resulting individual gene phylogenies were submitted to the CLANN software [104] to generate the supertree using a heuristic search of the supertree space for identifying the best tree. The nearest neighbor-interchange and subtree pruning and regrafting methods were tested starting with a neighbor-joining tree calculated from the average consensus distances. A bootstrap analysis was performed using 100 replicates. Then, these individual phylogenies were compared with the phylogenomic tree obtained from the 2,684 alignment files, which was considered to represent the evolutionary history of the analyzed fungi. The aim was to evaluate which secretome proteins have an evolutionary pattern that is not compatible with the organism’s evolution.

During the submission process of this work, the genome sequence from another strain of *M. anisopliae* (Ma69) was accepted for publication [105]. Altogether, the genomic sequences of *Metarhizium* spp. will allow a deeper analysis of ancient mechanisms of virulence by entomopathogens.

### Sequence submission

The *M. anisopliae* E6 genome sequence is deposited in NCBI under Accession JNNZ00000000. RNAseq reads were deposited in NCBI SRA under Bioproject accession PRJNA257269.

## Electronic supplementary material

Additional file 1: ***M. anisopliae*** **E6 predicted proteins that exhibit matches in the PHI (Pathogen-Host Interaction) database proteins.** (DOCX 34 KB)

Additional file 2: **The automated refined secretome prediction pipeline used in this work.** (TIFF 188 KB)

Additional file 3: **The primary functional categories and PFAM structures of the refined predicted fungal secretomes.** (DOC 57 KB)

Additional file 4: **Enrichment analysis performed with the GO annotations of the predicted fungal secretomes.** Comparisons were conducted with GO annotations of differente lifestyles to entomopathogens employing Fischer enrichment test of the Blast2GO software. (XLSX 46 KB)

Additional file 5: **Predicted GPI-Ps coded by the** ***M. anisopliae*** **E6 genome.** (DOCX 115 KB)

Additional file 6: **Comparative analysis of GPI-Ps coding genes among** ***Metarhizium*** **spp.** Each line represents an ortholog, as revealed by the bidirectional best-hit BLAST analysis. Shaded cells represent predicted proteins obtained in the pipeline used in this work to identify secreted proteins. Empty cells represent the lack of an ortholog. (XLSX 16 KB)

Additional file 7: **Proportion of gene duplications for sequences predicted as secreted and non-secreted.** (DOCX 118 KB)

Additional file 8: **RNAseq and alignment analysis statistics.** (DOCX 50 KB)

Additional file 9: **Principal component analysis of the expression patterns of the three distinct conditions analyzed.** (PNG 96 KB)

Additional file 10: **Log fold change (logFC) for selected** ***M. anisopliae*** **E6 loci.** C-48 h: Control condition with no infection; I-48 h: host cuticle 48 hours infection and I-144 h: host cuticle 144 hours infection. *no significant variation (FDR < 0.05 and logFC ≥ 1 or ≤ -1). (DOC 52 KB)

Additional file 11: **Complete edgeR output for the differential expression analysis.** (XLS 2 MB)

Additional file 12: **Fungal evolutionary history obtained through a phylogenomic approach using Maximum Likelihood Method.** Each internal branch indicates the percentage of times the corresponding cluster was found among the 1,000 intermediate trees. The scale bar represents substitutions of amino acids per site. (TIFF 143 KB)

Additional file 13: **Description of the proteins identified as potentially responsible for the differential evolution of the secretome and their expression status based on RNA sequencing.** (XLS 30 KB)
