# Supplementary material for: Comparative genome analysis of entomopathogenic fungi reveals a complex set of secreted proteins
Source: BMC Genomics. 2014 Sep 29;15:822. doi: 10.1186/1471-2164-15-822 (PMC4246632; doi:10.1186/1471-2164-15-822)
Supplement: Supplementary file 1 — Additional file 1: M. anisopliae E6 predicted proteins that exhibit matches in the PHI (Pathogen-Host Interaction) database proteins. (DOCX 34 KB) [file 12864_2014_6687_MOESM1_ESM.docx]

**Additional File 1:** *M. anisopliae* predicted proteins exhibiting matches to PHI (Pathogen-Host Interaction) database proteins.

| ***M.anisopliae* ID** | ***M. anisopliae* product** | **PHI ID** | **Pathogen** | **Protein product**  **(protein name)** | **Phenotype of mutant** | **Experimental Host** |
| --- | --- | --- | --- | --- | --- | --- |
| MANI2801 | Chitinase chi2 | 2388 | *Metarhizium anisopliae* | Chitinase (chi2) | Reduced virulence | *Dysdercus peruvianus* |
| MANI23390 | Histidine kinase 1 | 2323 | *Metarhizium robertsii* | Histidine kinase 1 (mhk1) | Reduced virulence | *Tenebrio molitor* |
| MANI26282 | Guanine nucleotide-binding protein alpha subunit | 251 | *Fusarium oxysporum* | Guanine nucleotide-binding protein alpha subunit (FGA1) | Reduced virulence | Tomato |
| MANI110816 | Mitogen activated protein kinase | 2253 | *Fusarium oxysporum* | MAP kinase (Fmk1) | Reduced virulence | Tomato |
| MANI28581 | Stress-activated MAP kinase | 2327 | *Gibberella zeae* | Mitogen-activated protein kinase (HOG1) | Reduced virulence | Wheat |
| MANI16498 | Calcineurin subunit B | 2109 | *Magnaporthe grisea* | Calcineurin subunit B (CNB) | Reduced virulence,  Loss of pathogenicity | Wheat, Barley |
| MANI5081 | Protein kinase gsk3 | 1200 | *Fusarium graminearum* | Protein kinase Gsk3 homolog | Reduced virulence | Wheat |
| MANI3477 | NADPH oxidase | 1035 | *Claviceps purpurea* | NADPH oxidase 1 (Cpnox1) | Reduced virulence | ? |
| MANI4896 | Superoxide dismutase | 330 | *Botrytis cinerea* | Superoxide dismutase [Cu-Zn] BcSOD1 | Reduced virulence | Bean |
| MANI28584 | Hypothetical protein | 395 | *Fusarium oxysporum* | G-protein alpha subunit (FGA2) | Loss of pathogenicity | Tomato |
| MANI8122 | Tubulin alpha chain | 2530 | *Aspergillus fumigatus* | Tubulin alpha-1 subunit (TUB1) | Loss of pathogenicity | Mouse |
| MANI28514 | Putative MAP kinase | 266 | *Fusarium graminearum* | MAP kinase (MGV1) | Loss of pathogenicity, Reduced virulence | Wheat,  Tomato |
| MANI111120 | Isocitrate lyase | 305 | *Magnaporthe grisea* | Isocitrate lyase (ICL1) | Reduced virulence | Rice |
| MANI109544 | Casein kinase I, putative | 1203 | *Fusarium graminearum* | Sc Yck1/2/3 homolog | Reduced virulence | Wheat |
| MANI15922 | RAB/GTPase | 339 | *Colletotrichum lindemuthianum* | Putative Rab/GTPase (CLPT1) | Reduced virulence | Bean |
| MANI9470 | ATP citrate lyase | 2387 | *Fusarium graminearum* | ATP citrate lyase (ACL2) | Loss of pathogenicity | Wheat |
| MANI1020 | Protein arginine N-methyltransferase 1 | 2351 | *Fusarium graminearum* | Arginine methyltransferase (AMT1) | Reduced virulence | Wheat, Maize |
| MANI24629 | Putative serine/threonine kinase | 393 | *Claviceps purpurea* | Serine/threonine kinase (CpCOT1) | Loss of pathogenicity | Rye |
| MANI16992 | Serine/threonine-protein kinase sid2 | 1187 | *Fusarium graminearum* | Sc Dbf2/Dbf20 homolog | Reduced virulence | Wheat |
| MANI25991 | Small GTPase Rho3 | 1061 | *Magnaporthe oryzae* | GTP-binding protein (MgRho3) | Loss of pathogenicity | Rice |
| MANI22865 | Cyclophilin | 277 | *Botrytis cinerea* | Cyclophilin A (BCP1) | Reduced virulence | Tomato, Bean |
| MANI2466 | Glutamine:fructose-6-phosphate amidotransferase | 2513 | *Aspergillus fumigatus* | Glucosamine-fructose-6-phosphate aminotransferase (GFA1) | Loss of pathogenicity | Mouse |
| MANI15599 | Chitin synthase export chaperone | 337 | *Fusarium oxysporum* | Chitin synthase export chaperone (CHS7) | Reduced virulence | Tomato |
| MANI24109 | Argininosuccinate lyase | 200 | *Fusarium oxysporum* | Argininosuccinate lyase (ARG1) | Reduced virulence | Tomato |
| MANI110055 | Malate synthase | 2267 | *Stagonospora nodorum* | Malate synthase (Mls1) | Loss of pathogenicity | Wheat |
| MANI4204 | Zinc knuckle domain-containing protein | 2189 | *Magnaporthe_grisea* | Zinc finger protein GIS2 (MoGIS2) | Reduced virulence | Rice,  barley |
| MANI24802 | Arsenite resistance protein Ars2 | 1348 | *Fusarium graminearum* | (GzC2H008) | Reduced virulence | Wheat |
| MANI18860 | Chitinase | 409 | *Beauveria_bassiana* | Chitinase (BbCHIT1) | Increased virulence (Hypervirulence) | Aphid |
| MANI5030 | Exosomal core protein CSL4 | 1606 | *Fusarium graminearum* | Uncharacterized protein  (GzOB047) | Reduced virulence | Wheat |
| MANI11258 | MAP kinase kinase EMK1 | 1179 | *Fusarium graminearum* | Sc_Ste7 homolog | Reduced virulence, Loss of pathogenicity | Wheat,  Tomato |
| MANI28205 | Kinase domain containing protein | 1214 | *Fusarium graminearum* | Uncharacterized protein  (FGSG_07816) | Reduced virulence | Wheat |
| MANI17339 | Class V chitin synthase | 1056 | *Colletotrichum_graminicola* | Chitin synthase C  (CgCHSV) | Reduced virulence | Maize |
| MANI16843 | MADS box protein | 2188 | *Magnaporthe_grisea* | MADS box protein (MoMCM1) | Reduced virulence, Loss of pathogenicity | Rice,  Barley |
| MANI15391 | Putative transcriptional regulator | 1366 | *Fusarium graminearum* | Uncharacterized protein  (GzCON7) | Reduced virulence | Wheat |
| MANI1866 | protein kinase SNF1 | 301 | *Fusarium_oxysporum* | Protein kinase SNF1 | Reduced virulence, Penetration defect | Cabbage,  *Arabidopsis thaliana* |
| MANI27848 | Cell division control protein | 1178 | *Fusarium graminearum* | Uncharacterized protein  (GzCON7) | Reduced virulence | Wheat |
| MANI13638 | Histone deacetylase phd1 | 2005 | *Magnaporthe_grisea* | Histone deacetylase complex protein (HOS2) | Loss of pathogenicity | Rice,  Barley |
| MANI4854 | Myosin type II heavy chain | 1648 | *Fusarium graminearum* | Uncharacterized protein  (GzWing020) | Reduced virulence | Wheat |
| MANI9382 | Multiprotein-bridging factor 1 | 1533 | *Fusarium graminearum* | Uncharacterized protein  (GzLam002) | Reduced virulence | Wheat |
| MANI19374 | Hypothetical protein | 1598 | *Fusarium graminearum* | Uncharacterized protein  (GzOB038) | Reduced virulence | Wheat |
| MANI19025 | Stalk rot protein | 1628 | *Fusarium graminearum* | Stalk rot protein (FgFSR1) | Reduced virulence | Wheat |
| MANI19740 | Zn(II)2Cys6 transcriptional activator | 1967 | *Fusarium graminearum* | Uncharacterized protein  (GzZC282) | Reduced virulence | Wheat |
| MANI14622 | Mst3-like protein kinase, putative | 1216 | *Fusarium graminearum* | Uncharacterized protein  (FGSG_06420) | Reduced virulence | Wheat |
| MANI8572 | cAMP-dependent protein kinase catalytic subunit | 341 | *Colletotrichum lagenarium* | Catalytic subunit of cAMP-dependent protein kinase | Loss of pathogenicity | Cucumber |
| MANI28842 | RacA | 2054 | *Magnaporthe grisea* | GTP-binding protein | Loss of pathogenicity | Rice,  Barley |
| MANI3272 | RNA polymerase II holoenzyme cyclin-like subunit | 2418 | *Fusarium graminearum* | Cyclin C-like CID1 | Reduced virulence | Wheat |
| MANI1796 | PRO1 protein | 1917 | *Fusarium graminearum* | Uncharacterized protein  (GzZC232) | Reduced virulence | Wheat |
| MANI23034 | rheb small monomeric GTPase RhbA | 317 | *Aspergillus fumigatus* | Ras homolog enriched in brain  (RHBA) | Reduced virulence | Mouse |
| MANI27102 | Cyclophilin A | 249 | *Magnaporthe oryzae* | Peptidyl-prolyl cis-trans isomerase (CYP1) | Reduced virulence | Rice |
| MANI112231 | Chitin Synthase1 | 2359 | Botrytis_cinerea | Chitin synthase class III  Bcchs3a | Reduced virulence | Bean,  Grapes |

*M. anisopliae* predicted protein versus PHI-proteins blast analysis used e-value 10^-5^ and ≥ 50 % coverage. This resulted in 2,396 proteins that matched PHI databases. After that, the results were filtered by considering only proteins that show over 70 % identity with *M. anisopliae* proteins. From the 94 proteins with >70 % identity, 50 exhibit “loss of pathogenicity or reduced virulence” as phenotype characteristics in mutant strains and these are displayed above. When more than one PHI-protein matched with the same *M. anisopliae* protein, only the hit with higher identity is shown.
