# Supplementary figures and images for: Comparative genome analysis of entomopathogenic fungi reveals a complex set of secreted proteins
Source: BMC Genomics. 2014 Sep 29;15:822. doi: 10.1186/1471-2164-15-822 (PMC4246632; doi:10.1186/1471-2164-15-822)

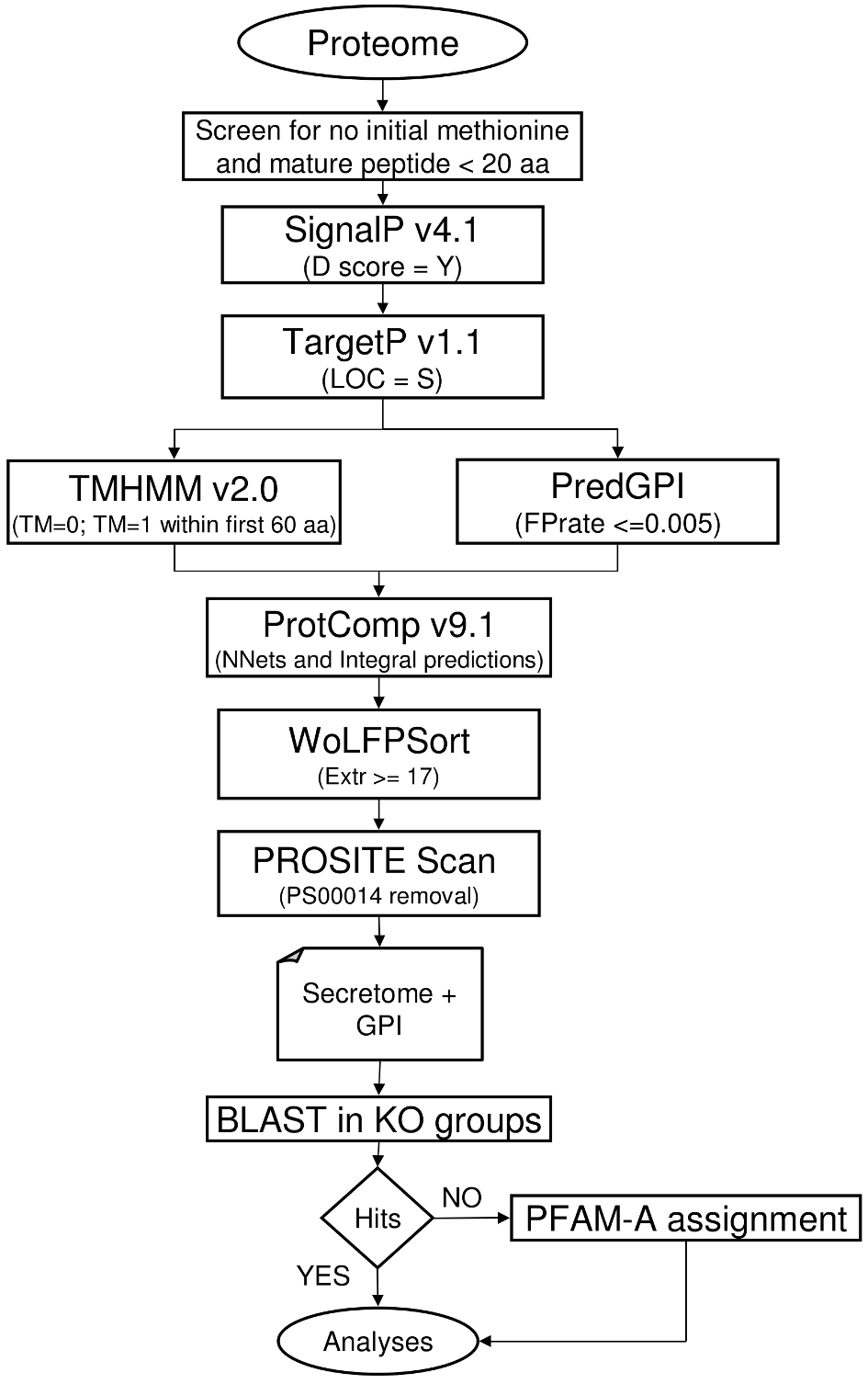

Supplement: Supplementary file 2 — Additional file 2: The automated refined secretome prediction pipeline used in this work. (TIFF 188 KB) [file 12864_2014_6687_MOESM2_ESM.tiff]

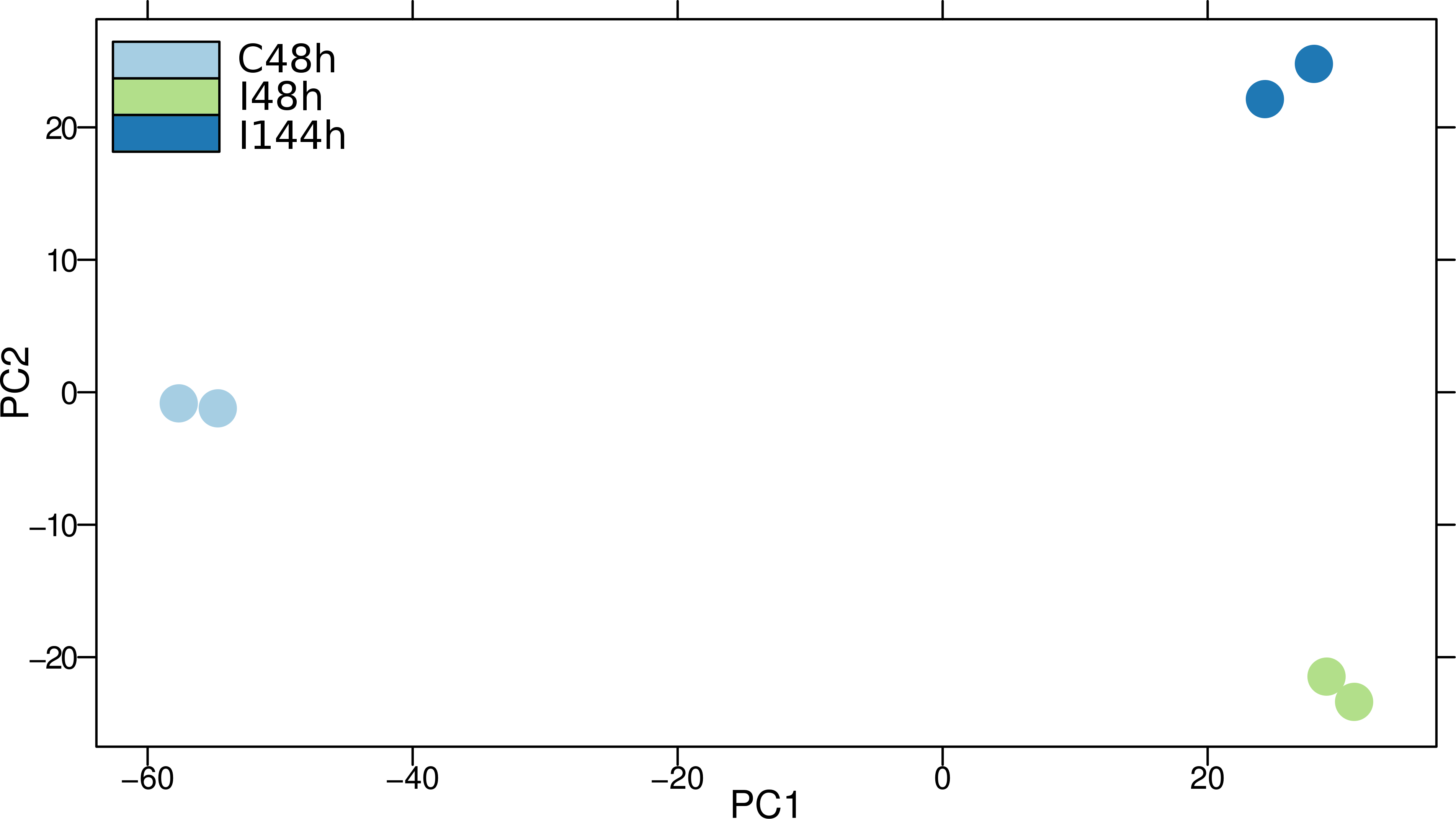

Supplement: Supplementary file 9 — Additional file 9: Principal component analysis of the expression patterns of the three distinct conditions analyzed. (PNG 96 KB) [file 12864_2014_6687_MOESM9_ESM.png]

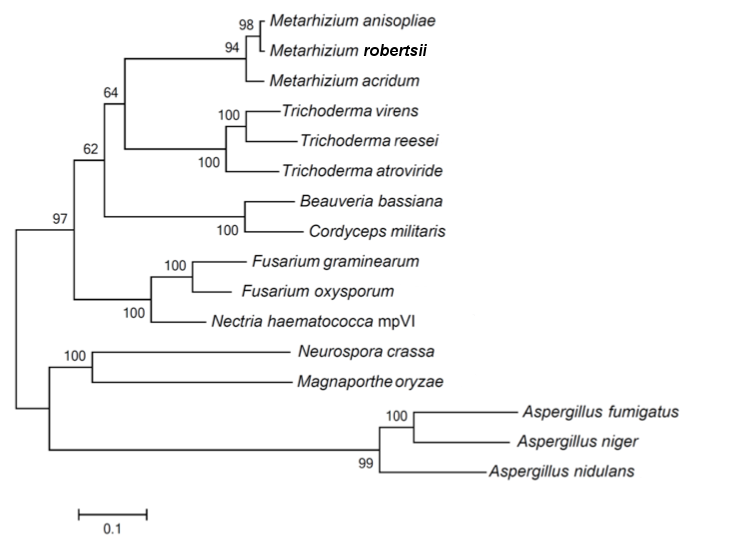

Supplement: Supplementary file 12 — Additional file 12: Fungal evolutionary history obtained through a phylogenomic approach using Maximum Likelihood Method. Each internal branch indicates the percentage of times the corresponding cluster was found among the 1,000 intermediate trees. The scale bar represents substitutions of amino acids per site. (TIFF 143 KB) [file 12864_2014_6687_MOESM12_ESM.tiff]
