# Supplementary material for: Comparative genome analysis of entomopathogenic fungi reveals a complex set of secreted proteins
Source: BMC Genomics. 2014 Sep 29;15:822. doi: 10.1186/1471-2164-15-822 (PMC4246632; doi:10.1186/1471-2164-15-822)
Supplement: Supplementary file 3 — Additional file 3: The primary functional categories and PFAM structures of the refined predicted fungal secretomes. (DOC 57 KB) [file 12864_2014_6687_MOESM3_ESM.doc]

Additional File 3: Refined fungi predicted secretomes main functional catergories and PFAM structures.

| Lifestyle | Entomopathogen | | | | | Human pathogen | | Saprophyte | | |
| --- | --- | --- | --- | --- | --- | --- | --- | --- | --- | --- |
| Organism | Mae6 | Mar | Mac | Com | Bba | Asf | Asni | Asn | Trr | Nec |
| Proteins predicted as secreted  (> 20 amino acids with initial M) | 405 (10777)  3.8% | 392 (10582)  3.7% | 308 (9849)  3.1% | 301 (9651)  3.1% | 391 (10364)  3.8% | 315 (9643)  3.3% | 409 (10573)  3.9% | 364 (10494)  3.5% | 296 (8140)  3.6% | 315 (9837)  3.2% |
| With GPI anchor  With BLAST hits  Hydrolase (EC:3.-.-.-)  Acting on ester bonds (EC:3.1.-.-)  Carboxylic-ester hydrolase (EC:3.1.1.-)  Phosphoric-diester hydrolase (EC:3.1.4.-)  Glycosidase (EC:3.2.1.-)  Chitinase (EC:3.2.1.14)  Peptidase (protease) (EC:3.4.-.-)  Aminopeptidase (EC:3.4.11.-)  Serine carboxypeptidases (EC:3.4.16.-)  Metallocarboxypeptidase (EC:3.4.17.-)  Serine endopeptidase (EC:3.4.21.-)  Trypsin (EC:3.4.21.4)  Aspartic endopeptidase (EC:3.4.23.-)  Metalloendopeptidase (EC:3.4.24.-)  Oxidoreductase (EC:1.-.-.-)  Others | 80  145  105  22  7  3  45 (167)  8 (30)  35  1 (65)  3 (11)  0 (5)  14 (65)  10 (17)  10 (35)  1 (21)  8  32 | 68  145  101  16  3  3  41 (164)  8 (30)  40  1 (54)  5 (11)  0 (4)  14 (78)  9 (27)  13 (33)  2 (25)  17  27 | 63  113  84  20  6  2  30 (142)  6 (21)  32  1 (54)  3 (13)  0 (4)  10 (51)  4 (10)  10 (27)  1 (17)  13  16 | 73  122  93  24  10  2  29 (146)  4 (25)  35  1 (42)  3 (13)  0 (8)  6 (39)  1 (7)  11 (33)  3 (25)  8  21 | 76  139  104  22  7  3  43 (151)  8 (22)  35  0 (41)  7 (13)  1 (7)  7 (53)  3 (12)  11 (32)  1 (27)  11  24 | 55  168  126  25  14  2  74 (227)  5 (20)  19  0 (54)  5 (12)  0 (5)  1 (22)  0 (0)  6 (12)  2 (19)  10  32 | 57  256  196  67  44  3  97 (248)  3 (23)  26  1 (63)  6 (11)  0 (6)  0 (25)  0 (0)  8 (17)  1 (26)  26  34 | 44  172  117  24  14  1  74 (215)  3 (21)  13  0 (58)  2 (5)  0 (6)  1 (19)  1 (1)  3 (13)  2 (18)  21  34 | 52  121  93  12  5  1  55 (152)  6 (19)  19  3 (40)  1 (6)  1 (7)  2 (0)  1 (1)  8 (18)  1 (16)  11  17 | 49  126  91  12  6  1  61 (155)  5 (15)  9  0 (37)  0 (6)  0 (5)  0 (16)  0 (0)  6 (22)  0 (15)  18  17 |
| With PFAM hits  Without BLAST hits  PFAM-Family  PF12296  PF01185  PF07249  PF01464  PF06766  PFAM-Domain  PF05730  PF09044  PF10528  PF00753 | 204  75  39  5  2  2  1  1  35  8  3  3  1 | 208  62  29  3  2  2  1  1  32  6  3  2  1 | 160  57  32  4  2  0  1  1  24  4  4  1  0 | 176  66  33  7  2  1  0  2  33  5  3  1  0 | 196  67  31  5  2  1  0  2  36  4  0  2  0 | 205  47  19  2  2  1  1  0  26  2  0  0  0 | 302  53  27  2  5  1  0  0  26  3  0  0  0 | 234  68  27  1  4  1  0  0  40  3  0  0  0 | 175  60  20  0  0  3  0  6  39  7  2  1  0 | 167  49  25  0  0  3  0  1  23  4  0  0  0 |

Additional File 3: Refined fungi predicted secretomes main functional catergories and PFAM structures (cont.).

| Lifestyle | Plant pathogen | | | | Mycoparasite | |
| --- | --- | --- | --- | --- | --- | --- |
| Organism | Fug | Fuo | Mao | Nhe | Tra | Trv |
| Proteins predicted as secreted  (> 20 amino acids with initial M) | 416 (11606)  3.6% | 579 (14880)  3.9% | 610 (12639)  4.8% | 472 (14791)  3.2% | 444 (11031)  (4.0% | 426 (11228)  3.8% |
| With GPI anchor  With BLAST hits  Hydrolase (EC:3.-.-.-)  Acting on ester bonds (EC:3.1.-.-)  Carboxylic-ester hydrolase (EC:3.1.1.-)  Phosphoric-diester hydrolase (EC:3.1.4.-)  Glycosidase (EC:3.2.1.-)  Chitinase (EC:3.2.1.14)  Peptidase (EC:3.4.-.-)  Aminopeptidase (EC:3.4.11.-)  Serine carboxypeptidases (EC:3.4.16.-)  Metallocarboxypeptidase (EC:3.4.17.-)  Serine endopeptidase (EC:3.4.21.-)  Trypsin (EC:3.4.21.4)  Aspartic endopeptidase (EC:3.4.23.-)  Metalloendopeptidase (EC:3.4.24.-)  Oxidoreductase (EC:1.-.-.-)  Others | 75  161  105  23  13  1  53 (227)  2 (22)  20  0 (98)  2 (11)  0 (11)  4 (28)  1 (1)  7 (21)  4 (26)  15  41 | 95  206  135  29  16  1  82 (310)  3 (29)  16  1 (104)  2 (18)  0 (10)  7 (50)  1 (1)  5 (23)  0 (22)  26  45 | 87  202  142  22  10  0  80 (238)  3 (24)  29  1 (89)  3 (10)  2 (10)  10 (42)  0 (0)  6 (24)  0 (19)  22  38 | 97  172  115  32  17  1  60 (280)  5 (26)  14  1 (106)  3 (23)  0 (11)  3 (63)  1 (1)  6 (22)  1 (16)  12  45 | 59  193  149  18  8  1  86 (210)  12 (30)  34  4 (58)  0 (9)  1 (8)  6 (48)  1 (1)  11 (24)  2 (21)  17  27 | 58  180  138  22  13  2  82 (227)  9 (38)  24  2 (72)  2 (7)  1 (7)  3 (42)  2 (2)  7 (23)  2 (22)  16  26 |
| With PFAM hits  Without BLAST hits  PFAM-Family  PF12296  PF01185  PF07249  PF01464  PF06766  PFAM-Domain  PF05730  PF09044  PF10528  PF00753 | 223  72  35  1  1  4  0  1  37  6  4  2  1 | 288  92  40  0  0  3  0  3  51  4  0  4  0 | 259  69  28  2  1  1  0  2  40  9  0  0  0 | 240  81  29  0  1  4  1  1  51  11  0  1  0 | 262  80  32  1  0  5  1  9  48  5  4  1  0 | 240  72  29  1  0  5  0  7  42  6  2  0  0 |

EC: Enzyme comission number; Numbers in parenthesis refers to complete proteome. PFAM identifiers are described in the text. Asf: *Aspergillus fumigatus*; Asn: *Aspergillus* *nidulans*; Asni: *Aspergillus* *niger*; Bba: *Beauveria* *bassiana*; Com: *Cordyceps* *militaris*; Fug: *Fusarium* *graminearum*; Fuo: *Fusarium* *oxysporum*; Mae6: *Metarhizium* *anisopliae* E6; Mar: *Metarhizium robertsii;* Mac: *Metarhizium* *acridum*; Mao: *Magnaporthe* *oryzae*; Nec: *Neurospora* *crassa*; Nhe: *Nectria* *haematococca*; Tra: *Trichoderma* *atroviride*; Trr: *Trichoderma* *reesei*; Trv: *Trichoderma* *virens*. * Proportion of duplicated secreted genes is statistically smaller than not secreted genes. Proportions test was conducted with R package (one-tailed prop.test).
