# Supplementary material for: Comparative genome analysis of entomopathogenic fungi reveals a complex set of secreted proteins
Source: BMC Genomics. 2014 Sep 29;15:822. doi: 10.1186/1471-2164-15-822 (PMC4246632; doi:10.1186/1471-2164-15-822)
Supplement: Supplementary file 5 — Additional file 5: Predicted GPI-Ps coded by the M. anisopliae E6 genome. (DOCX 115 KB) [file 12864_2014_6687_MOESM5_ESM.docx]

Additional File 5: Predicted GPI-Ps coded by *M. anisopliae* genome.

| **Sequence description** | **InterPro** | **Pfam** | **Pfam description** |
| --- | --- | --- | --- |
| MANI16246 glycosyl hydrolase, putative [EC:3.2.1.101] | IPR005198 | GH76 | Glycoside hydrolase family 76 |
| MANI28094 cell wall glycosyl hydrolase Dfg5, putative [EC:3.2.1.101] | IPR005198 | GH76 | Glycoside hydrolase family 76 |
| MANI6418 glycosyl hydrolase family 76 | IPR005198 | GH76 | Glycosyl hydrolase family 76 |
| MANI10680 GPI-anchored cell wall beta-1,3-endoglucanase EglC | IPR000490 | GH17 | Glycoside hydrolase family 17 |
| MANI17257 GPI-anchored cell wall beta-1,3-endoglucanase EglC | IPR000490 | GH17 | Glycoside hydrolase family 17 |
| MANI9409 beta-1,3-endoglucanase, putative |  | GH16 |  |
| MANI12787 cell wall glucanosyltransferase Mwg1 | IPR000757 | GH16 | Glycosyl hydrolases family 16 |
| MANI18580 Cell wall glucanosyltransferase Mwg1 [EC:2.4.1.-] | IPR000757 | GH16 | Glycoside hydrolase family 16 |
| MANI 118340 extracellular cell wall glucanase Crf1 | IPR000757 | GH16 | Glycosyl hydrolases family 16 |
| MANI19411 putative xylanase 3 | IPR000757 | GH16 | Glycoside hydrolase family 16 |
| MANI22365 glycoside hydrolase family 24 protein | IPR002196 | GH24 | Phage Lysozyme |
| MANI2103 secreted aspartic proteinase | IPR001461 | Asp | Eukaryotic aspartyl protease |
| MANI5501 candidapepsin-4 precursor | IPR001461 | Asp | Eukaryotic aspartyl protease |
| MANI21206 Cell wall galactomannoprotein domain-containig protein | IPR021054 | HsbA | Hydrophobic surface binding protein A |
| MANI13004 Cell wall galactomannoprotein [EC:3.2.1.14] | IPR021054 | HsbA | Hydrophobic surface binding protein A |
| MANI1120 Conserved hypothetical protein | IPR008427 | CFEM | CFEM Domain |
| MANI10995 hypothetical protein | IPR008427 | CFEM | CFEM Domain |
| MANI12030 conserved hypothetical protein | IPR008427 | CFEM | CFEM Domain |
| MANI1086 Conserved hypothetical protein | IPR008427 | CFEM | CFEM Domain |
| MANI18373 hypothetical protein | IPR008427 | CFEM | CFEM Domain |
| MANI1192 CFEM domain-containing protein, putative | IPR008427 | CFEM | CFEM Domain |
| MANI13188 Conserved hypothetical protein | IPR002889 | WSC | WSC domain |
| MANI10403 hypothetical protein | IPR002889 | WSC | WSC Domain |
| MANI1730 conserved hypothetical protein | IPR018466 | GPI-anchored | Ser-Thr-rich glycosyl-phosphatidyl-inositol-anchored membrane family |
| MANI1395 Extracellular matrix protein precursor | IPR018466 | GPI-anchored | Ser-Thr-rich glycosyl-phosphatidyl-inositol-anchored membrane family |
| MANI13738 putative acetyl xylanesterase | IPR000675 | Cutinase | Cutinase |
| MANI16631 Acid phosphatase [EC:3.1.3.2] | IPR004843 | Metallophos | Calcineurin-like phosphoesterase |
| MANI110476 lysophospholipase precursor [EC:3.1.1.5] | IPR002642 | PLA2 B | Lysophospholipase catalytic domain |
| MANI23073 putative stretch-activated Ca channel | IPR024338 | Mid1 | Stretch-activated Ca2+-permeable channel component |
| MANI8382 Covalently-linked cell wall protein | IPR000420 | PIR | Yeast PIR protein repeat |
| MANI20272 hypothetical protein | IPR025332 | DUF4243 | Protein of unknown function |
| MANI9626 DUF2183 domain-containing protein | IPR019236 | DUF2183 | Uncharacterized conserved protein |
| MANI9545 Cupredoxin, putative | IPR008972 |  |  |
| MANI9357 PLC-like phosphodiesterase, putative | IPR017946 |  |  |
| MANI6442 PLC-like phosphodiesterase, putative | IPR017946 |  |  |
| MANI465 Conserved hypothetical protein |  |  |  |
| MANI14934 prp 4 CRoW domain-containing protein |  |  |  |
| MANI6763 phosphoglycerate mutase family protein |  |  |  |
| MANI680 Extracellular serine-rich protein |  |  |  |
| MANI24244 Mmc protein putative |  |  |  |
| MANI13411 clock-controlled protein, putative |  |  |  |
| MANI1520 Conserved hypothetical protein |  |  |  |
| MANI1545 Conserved hypothetical protein |  |  |  |
| MANI2999 hypothetical protein |  |  |  |
| MANI4480 Conserved hypothetical |  |  |  |
| MANI5253 Conserved hypothetical |  |  |  |
| MANI7716 Hypothetical protein |  |  |  |
| MANI10971 hypothetical protein |  |  |  |
| MANI11622 unnamed protein product |  |  |  |
| MANI16248 conserved hypothetical protein |  |  |  |
| MANI16784 Conserved hypothetical protein |  |  |  |
| MANI21499 hypothetical protein |  |  |  |
| MANI22607 hypothetical protein |  |  |  |
| MANI22693 Conserved hypothetical protein |  |  |  |
| MANI110571 unnamed protein product |  |  |  |
| MANI7808 Conserved hypothetical protein |  |  |  |
| MANI548 Hypothetical protein |  |  |  |
| MANI8923 Hypothetical protein |  |  |  |
| MANI22032 hypothetical protein |  |  |  |
| MANI1481 Conserved hypothetical protein |  |  |  |
| MANI4908 conserved hypothetical |  |  |  |
| MANI5419 conserved hypothetical protein |  |  |  |
| MANI9122 Hypothetical protein |  |  |  |
| MANI9209 Hypothetical protein |  |  |  |
| MANI9360 Hypothetical protein |  |  |  |
| MANI9379 Conserved hypothetical protein |  |  |  |
| MANI10758 hypothetical protein |  |  |  |
| MANI13786 hypothetical protein |  |  |  |
| MANI13980 hypothetical protein |  |  |  |
| MANI15787 conserved hypothetical protein |  |  |  |
| MANI16814 Conserved hypothetical protein |  |  |  |
| MANI16816 Conserved hypothetical protein |  |  |  |
| MANI18605 hypothetical protein |  |  |  |
| MANI 120950 hypothetical protein |  |  |  |
| MANI24329 conserved hypothetical protein |  |  |  |
| MANI22661 conserved glycine-rich protein |  |  |  |
| MANI109739 unnamed protein product |  |  |  |
| MANI111160 unnamed protein product |  |  |  |
| MANI24337 hypothetical protein |  |  |  |
| MANI20960 hypothetical protein |  |  |  |
| MANI16246 glycosyl hydrolase, putative [EC:3.2.1.101] | IPR005198 | GH76 | Glycoside hydrolase family 76 |
| MANI28094 cell wall glycosyl hydrolase Dfg5, putative [EC:3.2.1.101] | IPR005198 | GH76 | Glycoside hydrolase family 76 |
