# Supplementary material for: Comparative genome analysis of entomopathogenic fungi reveals a complex set of secreted proteins
Source: BMC Genomics. 2014 Sep 29;15:822. doi: 10.1186/1471-2164-15-822 (PMC4246632; doi:10.1186/1471-2164-15-822)
Supplement: Supplementary file 7 — Additional file 7: Proportion of gene duplications for sequences predicted as secreted and non-secreted. (DOCX 118 KB) [file 12864_2014_6687_MOESM7_ESM.docx]

**Additional File 7:** Proportion of gene duplications for sequences predicted as secreted and not secreted.

| **Organism** | **Secreted** | | **Not secreted** | | **P-value**  **Proportions** | **Proteome duplication (%)** |
| --- | --- | --- | --- | --- | --- | --- |
|  | **Single copy** | **>=2 copies (%)** | **Single copy** | **>=2 copies Count (%)** |  |  |
| **Asf** | 277 | 38 (12.1%) | 8753 | 575 (6.2%) | **0.000** | 6.4 |
| **Asn** | 303 | 61 (16.8%) | 9392 | 778 (7.7%) | **0.000** | 8.0 |
| **Asni** | 309 | 100 (24,4%) | 8854 | 1310 (12.9%) | **0.000** | 13.3 |
| **Bba** | 348 | 43 (11.0%) | 9113 | 860 (8.6%) | 0.062 | 8.7 |
| **Com** | 280 | 21 (7.0%) | 8890 | 460 (4.9%) | 0.069 | 5.0 |
| **Fug** | 389 | 27 (6,5%) | 10573 | 617 (5.5%) | 0.228 | 5.5 |
| **Fuo** | 505 | 74 (12.8%) | 12743 | 1558 (10.9%) | 0.088 | 11.0 |
| **Mae6** | 379 | 26 (6.4%) | 9752 | 620 (6.0%) | 0.500 | 6.0 |
| **Mar** | 363 | 29 (7.4%) | 9517 | 673 (6.6%) | 0.303 | 6.6 |
| **Mac** | 288 | 20 (6.5%) | 9011 | 530 (5.6%) | 0.281 | 5.6 |
| **Mao** | 479 | 131 (21.5%) | 10646 | 1383 (11.5%) | **0.000** | 12.0 |
| **Nec** | 288 | 27 (8.6%) | 9066 | 456 (4.8%) | **0.002** | 4.9 |
| **Nhe*** | 421 | 51 (10.8%) | 12240 | 2079 (14.5%) | **0.024** | 14.4 |
| **Tra** | 387 | 57 (12.8%) | 10049 | 538 (5.1%) | **0.000** | 5.4 |
| **Trr** | 279 | 17 (5.7%) | 7556 | 288 (3.7%) | **0.046** | 3.7 |
| **Trv** | 382 | 44 (10.3%) | 10091 | 711 (6.6%) | **0.002** | 6.7 |

Asf: *Aspergillus fumigatus*; Asn: *Aspergillus* *nidulans*; Asni: *Aspergillus* *niger*; Bba: *Beauveria* *bassiana*; Com: *Cordyceps* *militaris*; Fug: *Fusarium* *graminearum*; Fuo: *Fusarium* *oxysporum*; Mae6: *Metarhizium* *anisopliae* E6; Mar: *Metarhizium robertsii;* Mac: *Metarhizium* *acridum*; Mao: *Magnaporthe* *oryzae*; Nec: *Neurospora* *crassa*; Nhe: *Nectria* *haematococca*; Tra: *Trichoderma* *atroviride*; Trr: *Trichoderma* *reesei*; Trv: *Trichoderma* *virens*. * Proportion of duplicated secreted genes is statistically smaller than not secreted genes. Proportions test was conducted with R package (one-tailed prop.test).
