# Supplementary material for: Comparative genome analysis of entomopathogenic fungi reveals a complex set of secreted proteins
Source: BMC Genomics. 2014 Sep 29;15:822. doi: 10.1186/1471-2164-15-822 (PMC4246632; doi:10.1186/1471-2164-15-822)
Supplement: Supplementary file 8 — Additional file 8: RNAseq and alignment analysis statistics. (DOCX 50 KB) [file 12864_2014_6687_MOESM8_ESM.docx]

RNAseq alignment statistics.

| Sample | Raw reads | Aligned with Tophat2 | Aligned to protein coding genes (%) |
| --- | --- | --- | --- |
| C48-I | 13,776,211 | 7,192,033 | 311,510 (4.3%) |
| C48-II | 13,387,306 | 7,456,455 | 251,438 (3.4%) |
| I48-I | 18,401,144 | 10,597,188 | 381,452 (3.6%) |
| I48-II | 11,770,175 | 7,211,653 | 317,494 (4.4%) |
| I144-I | 11,195,513 | 4,625,009 | 420,346 (9.1%) |
| I144-II | 10,872,026 | 4,951,121 | 267,735 (5.4%) |
