# Supplementary material for: Comparative genome analysis of entomopathogenic fungi reveals a complex set of secreted proteins
Source: BMC Genomics. 2014 Sep 29;15:822. doi: 10.1186/1471-2164-15-822 (PMC4246632; doi:10.1186/1471-2164-15-822)
Supplement: Supplementary file 10 — Additional file 10: Log fold change (logFC) for selected M. anisopliae E6 loci. C-48 h: Control condition with no infection; I-48 h: host cuticle 48 hours infection and I-144 h: host cuticle 144 hours infection. *no significant variation (FDR < 0.05 and logFC ≥ 1 or ≤ -1). (DOC 52 KB) [file 12864_2014_6687_MOESM10_ESM.doc]

**Additional File 10**: Log Fold Change (logFC) for selected *M. anisopliae* loci. C48h: Control condition with no infection; I48h: host cuticle 48 hours infection and I144h: host cuticle 144 hours infection. * no significant variation (FDR < 0.05 and logFC ≥ 1 or ≤ -1).

| **Locus** | **logFC C48h x I48h** | | **logFC I48h x I144h** | | **Description** | |  |
| --- | --- | --- | --- | --- | --- | --- | --- |
| MANI10000 | | 3.9 | | -3.6 | | Subtilisin PR1J | |
| MANI21586 | | 2.5 | | -4.5 | | Subtilisin PR1C | |
| MANI29315 | | 5.0 | | -3.3 | | Subtilisin PR1I | |
| MANI3773 | | 9.2 | | -3.2 | | Subtilisin PR1K | |
| MANI19956 | | 3.4 | | -2.9 | | CAS1 appressorium specific protein | |
| MANI17853 | | 3.3 | | -4.4 | | Cell surface protein (Mas1) | |
| MANI8633 | | 2.6 | | -2.7 | | Cell surface protein (Mas1) | |
| MANI9461 | | * | | 3.3 | | Arginyl-tRNA synthetase | |
| MANI21563 | | * | | 2.2 | | Isoleucyl-tRNA synthetase | |
| MANI122550 | | 1.5 | | 2.5 | | Glutaminyl-tRNA synthetase | |
| MANI10008 | | * | | 2.4 | | Ribosome biogenesis protein Ria1 | |
| MANI14555 | | -1.4 | | 2.3 | | Serine/threonine-protein kinase RIO1 | |
| MANI9142 | | * | | -1.2 | | Glyceraldehyde 3-phosphate dehydrogenase | |
| MANI10753 | | * | | -1.3 | | Triosephosphate isomerase | |
| MANI2103 | | * | | -1.1 | | ATP citrate synthase | |
| MANI19967 | | 6.1 | | -5.6 | | Trypsin | |
| MANI13010 | | 2.9 | | -7.7 | | Trypsin | |
| MANI12760 | | -6.3 | | * | | Trypsin | |
| MANI13004 | | -5.1 | | * | | Aspartic endopeptidase | |
| MANI13481 | | -7.6 | | * | | Aspartic endopeptidase | |
| MANI17353 | | 1,8 | | * | | Aspartic endopeptidase | |
| MANI5230 | | 5.3 | | -2.3 | | Aspartic endopeptidase | |
| MANI18952 | | * | | 2.2 | | Aspartic endopeptidase | |
| MANI12831 | | 3,5 | | 1,3 | | Putative aspartic endopeptidase | |
| MANI6661 | | 1,2 | | * | | Chitinase | |
| MANI10895 | | 5.1 | | -2.4 | | Chitinase | |
| MANI10603 | | 1.3 | | * | | Chitin synthase | |
| MANI18943 | | -10.5 | | * | | Nitrate reductase | |
| MANI28221 | | 8.7 | | -6.4 | | Starvation-stress gene A | |
| MANI5169 | | 12.3 | | -1.7 | | Methyltransferase | |
| MANI10000 | | 3.2 | | * | | Superoxide dismutase | |
| MANI21586 | | 3.3 | | -3.2 | | Peroxisomal catalase | |
| MANI29315 | | 2.7 | | -2.2 | | Catalase-peroxidase | |
| MANI3773 | | 7.9 | | -4.1 | | Glutathione S-transferase | |
| MANI19956 | | 10.2 | | -1.8 | | Glutathione S-transferase | |
| MANI17853 | | -2.4 | | * | | Thioredoxin reductase | |
| MANI8633 | | -4.9 | | -1.5 | | Peroxiredoxin | |
